# Genome-Wide Association Study (GWAS) and genome prediction of seedling salt tolerance in bread wheat (*Triticum aestivum* L.)

**DOI:** 10.1186/s12870-022-03936-8

**Published:** 2022-12-13

**Authors:** Saeideh Javid, Mohammad Reza Bihamta, Mansour Omidi, Ali Reza Abbasi, Hadi Alipour, Pär K. Ingvarsson

**Affiliations:** 1 Department of Agronomy and Plant Breeding, University of Tehran, Karaj, Iran; 2 Department of Plant Production and Genetics, Urmia University, Urmia, Iran; 3 Department of Plant Biology, Swedish University of Agricultural Sciences, Uppsala, Sweden

**Keywords:** Association mapping, Abiotic stress, Genomic selection, Genotyping-by-sequencing; Salinity stress, Seedling, Wheat accessions

## Abstract

**Background:**

Salinity tolerance in wheat is imperative for improving crop genetic capacity in response to the expanding phenomenon of soil salinization. However, little is known about the genetic foundation underlying salinity tolerance at the seedling growth stage of wheat. Herein, a GWAS analysis was carried out by the random-SNP-effect mixed linear model (mrMLM) multi-locus model to uncover candidate genes responsible for salt tolerance at the seedling stage in 298 Iranian bread wheat accessions, including 208 landraces and 90 cultivars.

**Results:**

A total of 29 functional marker-trait associations (MTAs) were detected under salinity, 100 mM NaCl (sodium chloride). Of these, seven single nucleotide polymorphisms (SNPs) including rs54146, rs257, rs37983, rs18682, rs55629, rs15183, and rs63185 with R^2^ ≥ 10% were found to be linked with relative water content, root fresh weight, root dry weight, root volume, shoot high, proline, and shoot potassium (K^+^), respectively. Further, a total of 27 candidate genes were functionally annotated to be involved in response to the saline environment. Most of these genes have key roles in photosynthesis, response to abscisic acid, cell redox homeostasis, sucrose and carbohydrate metabolism, ubiquitination, transmembrane transport, chromatin silencing, and some genes harbored unknown functions that all together may respond to salinity as a complex network. For genomic prediction (GP), the genomic best linear unbiased prediction (GBLUP) model reflected genetic effects better than both bayesian ridge regression (BRR) and ridge regression-best linear unbiased prediction (RRBLUP), suggesting GBLUP as a favorable tool for wheat genomic selection.

**Conclusion:**

The SNPs and candidate genes identified in the current work can be used potentially for developing salt-tolerant varieties at the seedling growth stage by marker-assisted selection.

**Supplementary Information:**

The online version contains supplementary material available at 10.1186/s12870-022-03936-8.

## Background

Common wheat (*Triticum aestivum* L.) provides nearly 20% of the global supply of calories and carbohydrates for human consumption [1, 2]. The productivity of this crop is challenged by several threats like human activities, climatic change, and unfavorable environmental conditions [3, 4]. Soil salinity is one of the effects of climate change on the environment. The second biggest abiotic factor affecting agricultural productivity worldwide is salinity/salt stress, which damages numerous physiological, biochemical, and molecular processes [5, 6]. Salinity is one of the important abiotic stresses that can seriously disrupt wheat production [7]. Generally speaking, when neutral soluble salts (chlorine, calcium, sodium, etc.) excessively accumulate in the rhizosphere, they can disrupt nutrient uptake [8]. Therefore, excess salts in the soil can lead to nutrient imbalance and ionic toxicity/deficiency, which negatively affect wheat yield [8–10]. Previous studies have demonstrated yield loss of up to 50% in wheat when exposed to a high salt concentration [11]. Thus, there is a demand to uncover salinity-responsive genes and use them to develop new salt-tolerant varieties [12].

Salt tolerance is a complex trait that includes a variety of genes, regulation networks, signal transductions, and metabolic pathways [13–16]. On the other, wheat response to saline environments depends on the duration and intensity of the stress and differs between genotypes as well as growth stages [17, 18]. For these reasons, assessing a genetic panel for salt tolerance at the seedling growth stage is a difficult task for wheat breeders. To make further progress in the development of salinity-tolerant wheat varieties it is crucial to get a better understanding of the molecular basis of salinity tolerance-related traits by using genetic tools, like quantitative trait loci (QTL) mapping [19].

QTL mapping has been used for detecting genes/genomic regions linked to salt tolerance traits, such as bio-physiological (e.g., Na^+^/K^+^ ratio) and agronomical traits (e.g., grain yield) in the salt-stressed wheat fields [7, 15, 19]. Importantly, these endeavors have relied on mapping populations of small size and a low number of SSRs markers, reflecting a limited resolution of QTLs, which cannot be reliably adopted in the marker-assisted selection. In contrast, genome-wide association study (GWAS) provides an alternative to QTL mapping for identifying genes linked to the phenotype of interest [20]. Association mapping can be performed by single-locus (GLM and MLM) or multi-locus (mrMLM) models [21]. GLM and MLM models adopt a genome scan by testing SNP markers at a time and need strict multiple test correction (e.g., Bonferroni) for managing false positives. However, this process is often too conservative and may lead to the loss of statistical power, failing to detect true associations that may be important. Moreover, single-locus models cannot simultaneously estimate all marker effects, and thereby cannot present a proper model for complex traits, which are controlled by the cumulative effect of several genes. To overcome these challenges, multi-locus approaches have started to be widely adopted as an alternative approach for dissecting the molecular basis of quantitative traits in plants and crops [22–49].

Previous studies presented experimental evidence regarding the QTLs/ candidate genes related to the salt tolerance at the seedling stage (i.e., seedling salt tolerance) in various plants/crops. In a research attempt, Luo et al. [30] elucidated the genetic basis of seedling salt tolerance by 557,894 polymorphic SNPs on 348 maize inbred lines. They identified 13 candidate genes associated with seedling salt tolerance by GWAS, among which, *ZmPMP3* and *ZmCLCg* were confirmed as genes involved in seedling salt tolerance. Interestingly, *ZmCLCg* was found as a chloride transport in maize. By using 18,430 polymorphic SNPs on 149 cotton genotypes, Zheng et al. [7] found six seedling salt tolerance genes, including *Gh_D08G1309*, *Gh_D08G1308*, *Gh_A01G0908*, *Gh_A01G0906*, *Gh_D01G0945*, and Gh*_D01G0943*, which were found to be responsible for cell amplification, auxin response, N-glycosylation, transmembrane transport, osmotic pressure balance, sucrose synthesis, and intracellular transport, respectively. Thabet et al. [28] evaluated 121 barley accessions for seedling salt tolerance by using 9 K SNPs and revealed around 1500 candidate genes, which encode potassium channels mapped on Ch.1H. The squamosa promoter-binding-like protein 6 at Ch.5H was detected to be linked with seedling salt tolerance. Screening a total of 203 rice accessions led to uncovering of 26 QTLs for seedling salt tolerance. Candidate genes for promising QTLs included glycosyl hydrolase, sucrose transporter, leucine zipper TF, ammonium transporter, and MYB TF [48].

As auxiliary tools for GWAS, genomic prediction boosts the speed and effectiveness of breeding by decreasing the time required for breeding cycles and by increasing selection accuracy [23]. The marker set, genomic selection method, population structure, and trait genetic architecture are the main factors that impact genomic accuracy. Several projects have demonstrated moderate to high genomic accuracy for complex traits in barley [24], maize [25], oat [26], rice [27], and wheat [23]. However, genomic prediction of the salt tolerance at the seedling stage has not been reported in wheat.

To the best of our knowledge, little is known about genomic regions associated with salt tolerance at the seedling stage in wheat. Therefore, we uncovered putative candidate genes and evaluated the genomic prediction accuracy of salt tolerance at the seedling stage using three methods for building a genomic selection model, namely GBLUP, RRBLUP, and BRR.

## Results

### Traits phenotyping

**Fig. 1:**
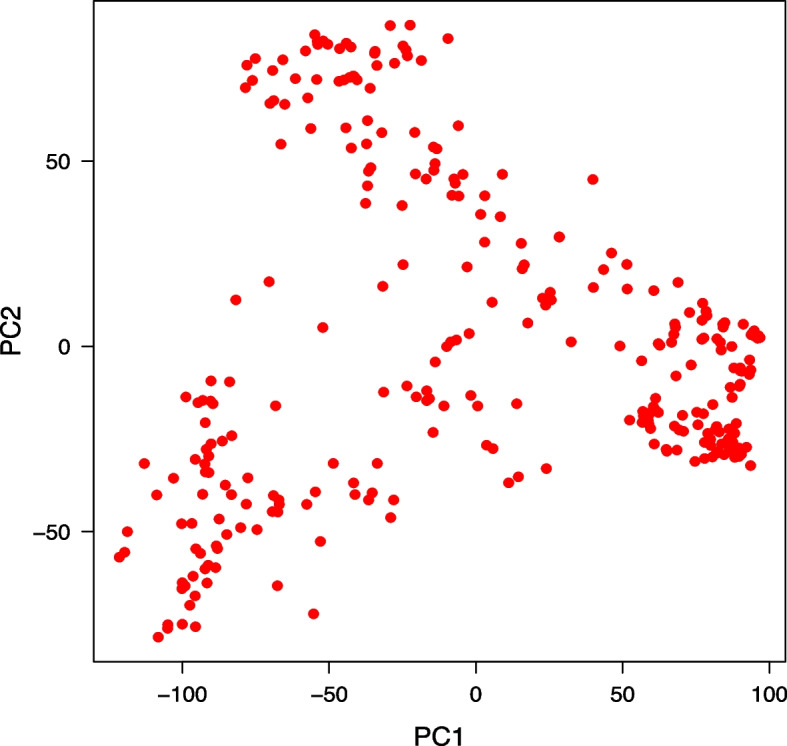
Principal component analysis for 298 Iranian bread wheat accessions (each red dot in the figure represents a genotype). PCA analysis, the estimated PCs showed that PCs 1 and 2 explained 17.0 and 6.4% of the genotypic variation, respectively

The phenotypic evaluation showed that most seedling-related traits have lower performance under salinity than normal conditions, highlighting salt stress limits seedling growth (Table 1). In the salt-stressed wheat, the K^+^/Na^+^ ratio in root and shoot exhibited nearly 53 and 33% decrease, respectively, reflecting these traits are highly sensitive to salinity. In contrast, salt stress led to an increase in some traits like ELI (6-fold), proline (7.8-fold), MDA (8.2-fold), and root volume (0.85-fold), suggesting that these traits are also strongly regulated by signals from salt stress (Table 2). From the perspective of the data desirability for GWAS analysis, a favorable range of variation coefficient (CV ≥ 10%) was observed for the seedling traits, except for root volume and MDA, under salt stress (Table 2). The highest CV was recorded for root K^+^ followed by total chlorophyll, root Na^+^, RWC, and SPAD, showing the potential of these traits to be used in selection-assisted breeding. The frequency distributions of seedling traits are displayed in Fig. 1S.

**Table 1:** The t-test for seedling-related traits of Iranian bread wheat accessions between normal and salinity conditions

Variables	Treatment	Mean	Std. Deviation	Std. Error	Mean Difference	t-test
ELI	Normal	6.5052	4.7037	0.2617	−14.588	−28.84**
	Stress	21.093	7.7813	0.4330		
SPAD	Normal	35.967	3.2659	0.1817	−7.607	−22.17**
	Stress	43.574	5.2312	0.2911		
SFW	Normal	14.520	1.6066	0.0894	4.8797	43.25**
	Stress	9.6406	1.2369	0.0688		
SDW	Normal	2.1574	0.3229	0.0180	0.7012	30.68**
	Stress	1.4562	0.2539	0.0141		
RWC	Normal	89.434	4.8421	0.2694	11.018	22.86**
	Stress	78.416	7.1816	0.3996		
RFW	Normal	8.9190	2.5019	0.1392	4.5329	28.61**
	Stress	4.3861	1.3587	0.0756		
RDW	Normal	1.4441	0.5354	0.0298	0.9829	31.45**
	Stress	0.4612	0.1701	0.0095		
RV	Normal	14.968	4.6072	0.2564	5.3483	16.36**
	Stress	9.6200	3.6486	0.2030		
SH	Normal	59.684	6.7591	0.3761	4.1262	8.289**
	Stress	55.558	5.8604	0.3261		
RH	Normal	39.415	5.4726	0.3045	7.0728	18.40**
	Stress	32.342	4.2177	0.2347		
Chl a	Normal	0.0236	0.0014	0.0001	0.0013	10.77**
	Stress	0.0223	0.0016	0.0001		
Chl b	Normal	0.0047	0.0006	0.0001	0.0004	7.368**
	Stress	0.0043	0.0007	0.0001		
Total Chl	Normal	0.0283	0.0012	0.0001	0.0017	16.08**
	Stress	0.0266	0.0014	0.0001		
Car	Normal	0.0648	0.0047	0.0003	0.0015	3.829**
	Stress	0.0633	0.0052	0.0003		
protein	Normal	13.055	1.0731	0.0597	1.8077	21.66**
	Stress	11.247	1.0476	0.0583		
proline	Normal	2.2568	0.3149	0.0175	−8.0829	−105.4**
	Stress	10.340	1.3412	0.0746		
CAT	Normal	0.0094	0.0010	0.0001	−0.0044	−40.72**
	Stress	0.0138	0.0017	0.0001		
GPX	Normal	0.1759	0.0391	0.0022	−0.0672	−17.14**
	Stress	0.2431	0.0586	0.0033		
MDA	Normal	3.4735	2.4921	0.1387	−8.6083	− 39.58**
	Stress	12.082	3.0116	0.1676		
Na-s	Normal	1202.5	397.97	22.144	− 2398.5	−41.54**
	Stress	3600.9	958.40	53.327		
Na-r	Normal	1366.6	159.57	8.8787	− 3437.8	−133.3**
	Stress	4804.3	435.16	24.213		
K-s	Normal	7842.5	889.07	49.469	239.35	3.259**
	Stress	7603.2	975.54	54.280		
K-r	Normal	6423.7	1029.1	57.262	−337.52	−5.856**
	Stress	6761.3	117.58	6.5423		
K/Na-s	Normal	7.5608	3.3085	0.1841	5.3262	28.57**
	Stress	2.2346	0.5302	0.0295		
K/Na-r	Normal	4.7981	0.8874	0.0494	3.6246	71.60**
	Stress	1.1734	0.2003	0.0111		

*Abbreviations*: *ELI* Electrolyte leakage, *SFW* SPAD; Shoot fresh weight, *SDW* Shoot dry weight, *RWC* Relative water content, *RFW* Root fresh weight, *RDW* Root dry weight, *RV* Root volume, *SH* Shoot height, *RH* Root height, *Chl a* Chlorophyll a, *Chl b* Chlorophyll b, *total Chl* Total chlorophyll, *Car* Carotenoid, *CAT* Protein; proline; catalase, *GPX* Guaiacol peroxidase, *MDA* Malondialdehyde, *Na-s* Shoot Na, *Na-r* Root Na, *K-s* Shoot K, *K-r* Root K, *K/Na-s* Shoot K/Na, *K/Na-r* root K/Na, *Std. Dev.* Standard deviation

**Table 2:** Descriptive statistics for seedling-related traits of Iranian bread wheat accessions under normal and salinity conditions

Normal						Sat stress				
Trait	Minimum	Maximum	Mean	Std. Dev.	CV (%)	Minimum	Maximum	Mean	Std. Dev.	CV (%)
ELI	1.66	38.72	6.51	4.70	72.19	21.09	7.78	46.25	6.74	14.57
SPAD	27.30	48.20	35.96	3.26	9.06	43.57	5.23	60.50	30.65	50.66
SFW	10.28	21.42	14.52	1.60	11.02	9.64	1.23	12.90	6.06	46.97
SDW	1.35	3.45	2.15	0.32	14.88	1.45	0.25	2.42	0.75	30.99
RWC	50.65	98.61	89.43	4.84	5.41	78.41	7.18	90.69	48.74	53.74
RFW	3.15	16.44	8.92	2.50	28.03	4.38	1.36	7.77	1.78	22.91
RDW	0.46	3.45	1.44	0.53	36.81	0.46	0.17	0.97	0.11	11.34
RV	5.30	32.20	14.96	4.61	30.82	9.62	3.65	27.55	2.0	7.25
SH	35.25	81.0	59.68	6.76	11.33	55.55	5.86	74.0	40.0	54.05
RH	26.0	58.50	39.41	5.47	13.88	32.34	4.22	46.0	22.5	48.91
Chl a	0.019	0.03	0.023	0.001	4.35	0.022	0.002	0.031	0.016	51.61
Chl b	0.002	0.007	0.004	0.0006	15	0.004	0.0006	0.0069	0.002	28.99
Total Chl	0.023	0.03	0.028	0.0012	4.28	0.026	0.0013	0.033	0.022	66.67
Car	0.048	0.097	0.064	0.0046	7.18	0.06	0.0052	0.094	0.045	47.87
protein	7.97	16.04	13.05	1.073	8.22	11.25	1.047	14.51	6.37	43.90
proline	0.93	3.16	2.25	0.314	13.95	10.34	1.34	19.93	7.44	37.33
CAT	0.007	0.014	0.009	0.0009	10	0.014	0.0016	0.024	0.009	37.50
GPX	0.095	0.49	0.17	0.03	17.65	0.24	0.058	0.49	0.124	25.31
MDA	1.119	24.57	3.47	2.49	71.76	12.08	3.01	31.94	3.12	9.77
Na-s	400.0	2900	1202	397.96	33.11	3601	958.40	8100	1500	18.52
Na-r	800.0	2000	1367	159.56	11.67	4804	435.15	6500	3500	53.85
K-s	5060	11,900	7843	889.06	11.34	7603	975.54	10,830	5060	46.72
K-r	2140	9910	6424	1029	16.02	6761	117.57	6770	4860	71.79
K/Na-s	2.93	29.50	7.56	3.31	43.78	2.23	0.53	5.07	0.77	15.19
K/Na-r	1.67	8.69	4.79	0.88	18.37	1.17	0.20	2.27	0.65	28.63

*Abbreviations*: *ELI* Electrolyte leakage, *SFW* SPAD; shoot fresh weight, *SDW* Shoot dry weight, *RWC* Relative water content, *RFW* Root fresh weight, *RDW* Root dry weight, *RV* Root volume, *SH* Shoot height, *RH* Root height, *Chl a* Chlorophyll a, *Chl b* Chlorophyll b, *total Chl* Total chlorophyll, *Car* Carotenoid, *CAT* Protein; proline; catalase, *GPX* Guaiacol peroxidase, *MDA* Malondialdehyde, *Na-s* Shoot Na, *Na-r* Root Na, *K-s* Shoot K, *K-r* Root K, *K/Na-s* Shoot K/Na, *K/Na-r* root K/Na, *Std. Dev.* Standard deviation

Pearson correlation coefficient analysis was used to assess the correlated responses to salt stress among different phenotypic traits. For example, root K^+^/Na^+^ ratio and root dry weight displayed a highly significant positive association (0.52) (*P* < 0.01) (Table 3).

**Table 3:** Correlation coefficients between the seedling-related traits for Iranian bread wheat accessions under normal (above the diameter) and salt stress (bottom the diameter) conditions

	ELI	SPAD	SFW	SDW	RWC	RFW	RDW	RV	SH	RH	Chl a	Chl b	Total Chl	Car	Protein	Proline	CAT	GPX	MDA	Na-s	Na-r	K-s	K-r	K/Na-s	K/Na-r
ELI	1	−.05	.11	.25**	−.13*	−.23**	−.17**	−.22**	.14*	−.18**	−.06	.01	−.07	−.07	−.01	−.01	−.04	−.00	.87**	−.06	.19**	−.01	.07	.08	−.08
SPAD	.10	1	−.05	.12*	.13*	−.21**	−.07	−.13*	−.15*	−.10	.04	.02	.05	.15**	−.04	.04	.07	.02	.03	.05	.07	−.21**	.23**	−.12*	.14
SFW	−.09	−.01	1	.57**	−.03	.21**	.33**	.37**	.13	.19**	−.07	−.06	−.11*	−.05	.08	.16**	−.15**	−.05	.10	−.17*	.28**	−.16	.31	.08	.09
SDW	.17**	.00	.57**	1	−.10	−.19**	−.05	−.07	.21	−.01	−.05	−.04	−.07	−.04	.05	.15**	−.13*	.04	.26**	−.12	.43**	−.22	.39	.05	.05
RWC	−.12*	.03	.14*	.09	1	.09	.04	.10	−.19	.08	.00	−.00	.00	.05	−.05	.00	.06	.00	−.12*	.07	−.05	−.01	−.01	−.03	.02
RFW	−.30**	.08	.43**	−.08	.12*	1	.79**	.71**	−.23	.26**	.01	−.04	−.01	.02	.09	−.07	−.06	−.04	−.2**	−.07	−.57**	.15**	−.17	.07	.29**
RDW	−.24**	.04	.31**	.03	.14**	.75**	1	.66**	−.16	.19**	.01	−.04	−.01	−.05	.10	.02	−.09	−.04	−.2**	−.15**	−.69**	.03	−.01	.07	.52**
RV	−.17**	.03	.27**	−.08	.09	.62**	.54**	1	−.12	.24**	−.04	.00	−.04	.00	.07	−.02	−.05	−.09	−.2**	−.16**	−.42**	−.06	.06	.09	.38
SH	.03	−.05	.09	.10	−.03	.11*	.09	.13*	1	−.07	.05	−.00	.06	−.01	−.05	−.19**	.03	−.01	.09	−.06	.20**	−.02	.06	.07	−.08
RH	−.13*	.06	.21**	−.04	.08	.36**	.25**	.46**	.10	1	−.03	.07	.01	.03	.04	.01	−.04	.01	−.12*	−.00	−.02	.02	−.01	−.07	.02
Chl a	−.00	−.02	.04	.05	−.02	.00	.03	−.01	−.03	−.03	1	−.4**	.88**	.03	.05	−.12*	−.06	.07	−.09	.11	−.02	.00	−.01	−.11	.03
Chl b	.06	.08	−.10	−.08	−.03	−.08	−.08	−.01	.03	−.03	−.53**	1	.01	.04	−.03	−.01	.08	−.02	.08	.02	.00	−.01	−.01	−.00	−.01
TotalChl	.03	.02	.00	.02	−.04	−.04	−.01	−.01	−.02	−.04	.91**	−.13*	1	.05	.04	−.14**	−.03	.07	−.06	.13*	−.02	−.00	−.01	−.13*	.02
Car	−.01	.04	−.01	−.01	.05	.01	.02	−.01	−.06	.02	−.04	.01	−.04	1	−.07	−.12*	.36**	.04	−.04	.121*	−.01	.02	−.04	−.14	−.04
Protein	−.06	−.01	.06	.00	.03	.12*	.08	.11*	.09	.06	.06	−.08	.04	.03	1	.01	−.79**	−.4**	−.02	−.05	−.01	.04	−.03	.09	−.01
Proline	.04	−.11	−.07	−.04	−.05	−.00	−.01	.02	.22**	.01	.01	.01	.01	.00	−.02	1	−.02	−.03	−.01	−.12*	.09	−.11	.15	.04	.05
CAT	.04	.06	−.09	−.04	−.03	−.12*	−.04	−.10	−.11*	−.06	−.07	.09	−.03	.36**	−.8**	.03	1	.26	−.00	.09	−.03	.04	−.06	−.11	−.05
GPX	−.00	.09	.05	.03	−.19**	−.04	−.04	.01	−.02	.00	.01	−.02	.00	−.00	−.4**	−.00	.34**	1	−.05	.03	−.02	−.06	.06	−.04	.06
MDA	−.26**	−.07	.21**	.04	.15**	.14**	.15**	.15**	−.14*	.12*	−.07	−.01	−.08	.02	−.08	−.04	.06	.04	1	−.01	.23**	−.01	.08	.05	−.11
Na-s	.041	−.08	−.24**	−.25**	.03	−.00	−.14**	−.07	−.07	−.02	−.04	−.03	−.06	−.06	−.05	.03	.05	−.04	−.02	1	.07	.21	−.22	−.73*	−.26*
Na-r	.01	−.08	.62**	.59**	.10	−.05	−.08	−.24**	.03	−.15**	.06	−.08	.03	.02	−.02	−.04	.00	.06	.04	−.25**	1	−.14	.23	−.04	−.5**
K-s	−.14*	−.00	.15**	−.05	.07	.38**	.34**	.27**	.22**	.15**	−.01	−.04	−.03	.10	−.02	.05	.05	.04	.02	.15**	−.11*	1	−.97	.06	−.73
K-r	.09	−.03	.11*	.19**	−.05	−.08	−.01	.03	.07	−.09	.06	−.08	.03	.01	−.01	.03	.06	−.02	.01	−.11	.14*	−.03	1	−.03	.69**
K/Na-s	−.08	.03	.26**	.16**	.01	.19**	.33**	.19**	.15**	.06	.01	.03	.03	.07	.07	.03	−.05	.01	.05	−.82**	.17**	.24**	.06	1	.01
K/Na-r	.12*	.03	−.28**	−.12*	−.06	−.26**	−.21**	.014	−.17**	.09	−.01	.08	.03	−.11*	.07	−.04	−.10	−.08	.01	−.04	−.39**	−.77**	−.05	−.24**	1

*Abbreviations*: *ELI* Electrolyte leakage, *SFW* SPAD; shoot fresh weight, *SDW* Shoot dry weight, *RWC* relative water content, *RFW* root fresh weight, *RDW* root dry weight, *RV* root volume, *SH* shoot height, *RH* root height, *Chl a* chlorophyll a, *Chl b* chlorophyll b, *total Chl* total chlorophyll, *Car* carotenoid, *CAT* protein; proline; catalase, *GPX* guaiacol peroxidase, *MDA* malondialdehyde, *Na-s* Shoot Na, *Na-r* Root Na, *K-s* Shoot K, *K-r* Root K, *K/Na-s* Shoot K/Na, *K/Na-r* root K/Na, *Std. Dev.* Standard deviation

### Marker distribution

Genotyping by sequencing a total of 298 Iranian bread wheat accessions yielded 566,439,207 unique reads. After alignment and de-duplication, 133,039 SNPs were called of which 10,938 had a MAF > 1%, heterozygosity< 10%, and missing data< 10%. These 10,938 SNPs were retained and used for the imputation process. The final data set included 46,203 imputed SNPs, which were used for subsequent association analyses.

### Linkage disequilibrium (LD)

In the panel of cultivars, LD calculation using 46,203 SNPs led to the detecting of 1,830,925 markers pairs (MPs), of which 60% of them displayed significant linkage. LD between marker pairs was recorded across the 21 chromosomes ranging from 0.14 (Ch.6D) to 0.37 (Ch.4A). The highest number of MPs were discovered in the B genome (949,425, 51.85%), followed by the A genome (675,325, 37%) and D genome (206,175, 11.26%) (Table 4).

**Table 4:** The SNP pairs as well as their LD (r^2^) and distance (cM) per chromosomes (Ch.) and genomes in Iranian bread wheat cultivars and landraces

Ch.	Cultivars				Landraces				Total			
	TNSP^a^	r^2^	Distance (cM)	NSSP^b^	TNSP^a^	r^2^	Distance (cM)	NSSP^b^	TNSP^a^	r^2^	Distance (cM)	NSSP^b^
1A	85,575	0.148218	1.7377	27,125 (31.7%)	92,925	0.112764	1.5964	33,515 (36.07%)	110,025	0.109029	1.3525	48,826 (44.38%)
2A	118,025	0.292156	0.9742	57,858 (49.02%)	123,175	0.297454	0.9444	68,675 (55.75%)	135,275	0.256551	0.8608	79,620 (58.86%)
3A	83,675	0.159365	2.5764	25,903 (30.96%)	73,525	0.136413	2.9397	28,144 (38.28%)	95,125	0.132082	2.2800	44,477 (46.76%)
4A	114,925	0.371766	1.5136	57,774 (50.27%)	108,375	0.376224	1.6121	65,451 (60.39%)	128,375	0.322641	1.3876	78,844 (61.42%)
5A	59,375	0.169369	2.3835	18,718 (31.53%)	58,475	0.150278	2.4165	24,007 (41.06%)	70,475	0.135122	2.0086	31,970 (45.36%)
6A	85,175	0.181387	1.4878	29,645 (34.8%)	84,425	0.181735	1.5010	40,176 (47.59%)	97,625	0.161099	1.2981	51,977 (53.24%)
7A	128,575	0.234215	1.3445	49,426 (38.44%)	126,575	0.214252	1.3660	63,357 (50.05%)	148,075	0.195064	1.1677	78,080 (52.73%)
1B	131,075	0.206251	1.0638	49,717 (37.93%)	133,525	0.157517	1.0413	63,803 (47.78%)	149,175	0.156549	0.9350	79,917 (53.57%)
2B	165,475	0.198105	0.8592	66,129 (39.96%)	155,625	0.177663	0.9135	78,536 (50.46%)	185,625	0.157919	0.7659	101,594 (54.73%)
3B	176,175	0.245726	0.8766	78,363 (44.48%)	170,925	0.221549	0.9040	89,150 (52.16%)	199,775	0.212639	0.7742	118,862 (59.5%)
4B	51,325	0.1455	2.5168	13,477 (26.26%)	43,025	0.1018	3.0028	12,311 (28.61%)	58,725	0.117756	2.2066	23,396 (39.84%)
5B	134,225	0.204683	1.4332	55,633 (41.45%)	134,675	0.14301	1.4493	56,285 (41.79%)	150,925	0.151374	1.2942	80,074 (53.06%)
6B	158,275	0.205457	0.7884	66,108 (41.77%)	164,475	0.139023	0.7587	71,582 (43.52%)	188,775	0.139448	0.6610	98,910 (52.4%)
7B	132,875	0.156677	1.1024	41,160 (30.98%)	125,875	0.129711	1.1575	50,573 (40.18%)	148,625	0.122897	0.9885	69,532 (46.78%)
1D	37,075	0.294821	4.4091	16,539 (44.61%)	40,975	0.232567	3.8321	19,755 (48.21%)	47,275	0.24563	3.4847	25,602 (54.16%)
2D	48,025	0.23446	2.2455	16,275 (33.89%)	52,825	0.169092	2.0486	20,548 (38.9%)	67,125	0.187305	1.6133	30,724 (45.77%)
3D	25,475	0.143085	6.2861	5413 (21.25%)	30,125	0.174879	5.3156	11,411 (37.88%)	35,525	0.128602	5.2147	10,004 (28.16%)
4D	10,275	0.167587	10.5662	2189 (21.3%)	10,375	0.14746	10.7135	3543 (34.15%)	12,125	0.1343	9.1793	4233 (34.91%)
5D	22,375	0.155406	9.3377	5503 (24.59%)	24,825	0.142184	8.3614	8953 (36.06%)	30,325	0.136465	6.9287	12,067 (39.79%)
6D	28,475	0.142966	5.3691	6844 (24.04%)	33,475	0.14123	4.5658	12,606 (37.66%)	36,875	0.12788	4.1511	15,587 (42.27%)
7D	34,475	0.208327	5.7957	10,809 (31.35%)	40,475	0.153099	4.9473	14,019 (34.64%)	44,975	0.155443	4.4523	17,504 (38.92%)
A genome	675,325	0.235213	1.6204	266,449 (39.45%)	667,475	0.223484	1.6427	323,325 (48.44%)	784,975	0.197227	1.4032	413,794 (52.71%)
B genome	949,425	0.20158	1.0837	370,587 (39.03%)	928,125	0.160951	1.1104	422,240 (45.49%)	1,081,625	0.156707	0.9550	572,285 (52.91%)
D genome	206,175	0.205106	5.3432	63,572 (30.83%)	233,075	0.170391	4.7074	90,835 (38.97%)	274,225	0.168573	4.1317	115,721 (42.2%)
Whole genome	1,830,925	0.214383	1.7613	700,608 (38.27%)	1,828,675	0.184979	1.6731	836,400 (45.74%)	2,140,825	0.173084	1.5262	1,101,800 (51.47%)

^a^ TNSP: Total number of SNP pairs; ^b^ NSSP: Number of significant SNP pairs (*P* < 0.001)

Implementing a similar test on wheat landraces led to uncovering 1,828,675 MPs with a mean r^2^ of 0.18, which is lower than that in wheat cultivars. Of course, a bigger part of marker pairs was found significant (836,400, 45.74%) in landraces. LD was strongest between marker pairs in Ch.4A (0.32), followed by Ch.2A (0.25) (Table 4).

### Population kinship and structure matrix

**Fig. 2:**
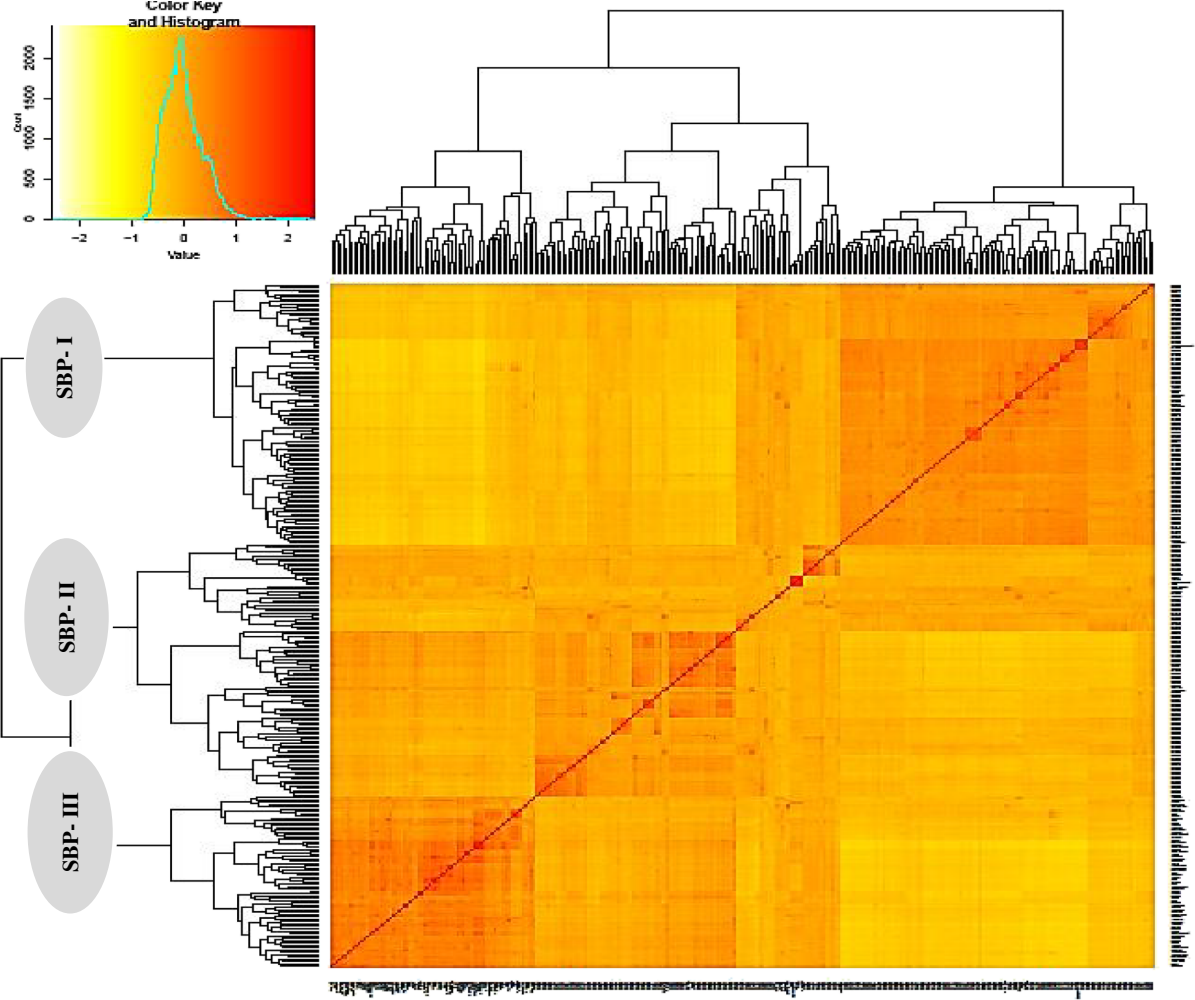
Kinship matrix-based cluster analysis for 298 Iranian bread wheat accessions reflecting three population substructures, Sub.1, Sub.2, and Sub.3. SBP-I subpopulation harbors 110 accessions (105 landraces and 5 cultivars), the SBP-II harbors 38 accessions (28 landraces and 10 cultivars), and the SBP-III harbors 144 accessions (69 landraces and 75 cultivars)

**Fig. 3:**
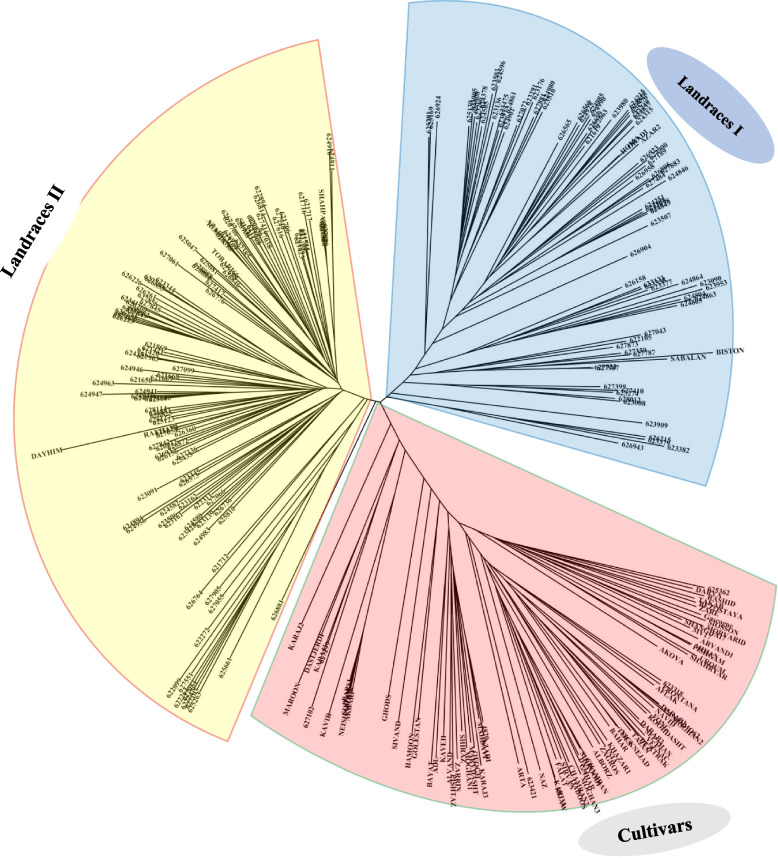
The dendrogram of Neighbor-Joining clustering constructed using 46,203 SNPs and 298 Iranian wheat accessions also clearly exhibited the clustering into three subgroups (landraces I, landraces II, and cultivars)

Based on the ∆K formula, the optimum number of subpopulations (K) in the association panel was estimated at K = 3 (Fig. 2S). From the PCA, first two PCs explained 17.0 and 6.4% of the genotypic variance, respectively (Fig. 1). Clear subpopulations were observed from the first two PCs, which indicated three subpopulations with admix accessions falling between clusters. As the panel of wheat cultivars and landrace have subpopulations, the PCA and kinship matrix were performed as variance-covariance. The cluster analysis based on the kinship matrix exhibited that the SBP-I subpopulation harbors 110 accessions (105 landraces and 5 cultivars), the SBP-II harbors 38 accessions (28 landraces and 10 cultivars), and the SBP-III harbors 144 accessions (69 landraces and 75 cultivars) (Fig. 2). A neighbor-joining tree of all accessions also clearly exhibited the clustering into three subgroups (Fig. 3).

### MTAs for seedling-related traits

**Fig. 4:**
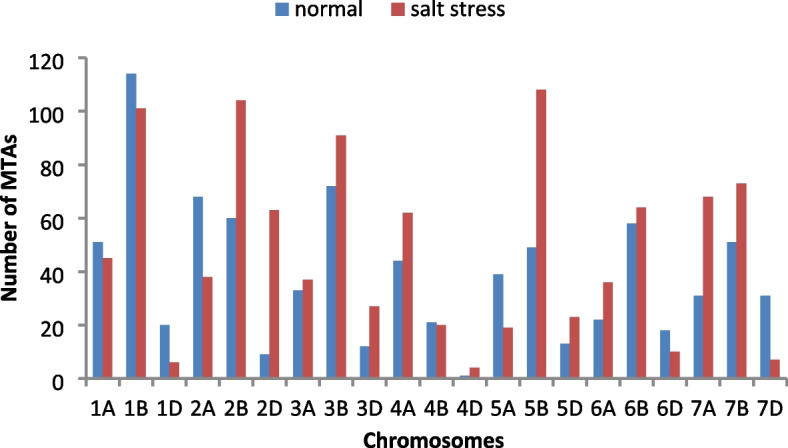
The number of marker-trait associations (MTAs) for seedling-related traits in Iranian bread wheat accessions under normal and salinity conditions

**Fig. 5:**
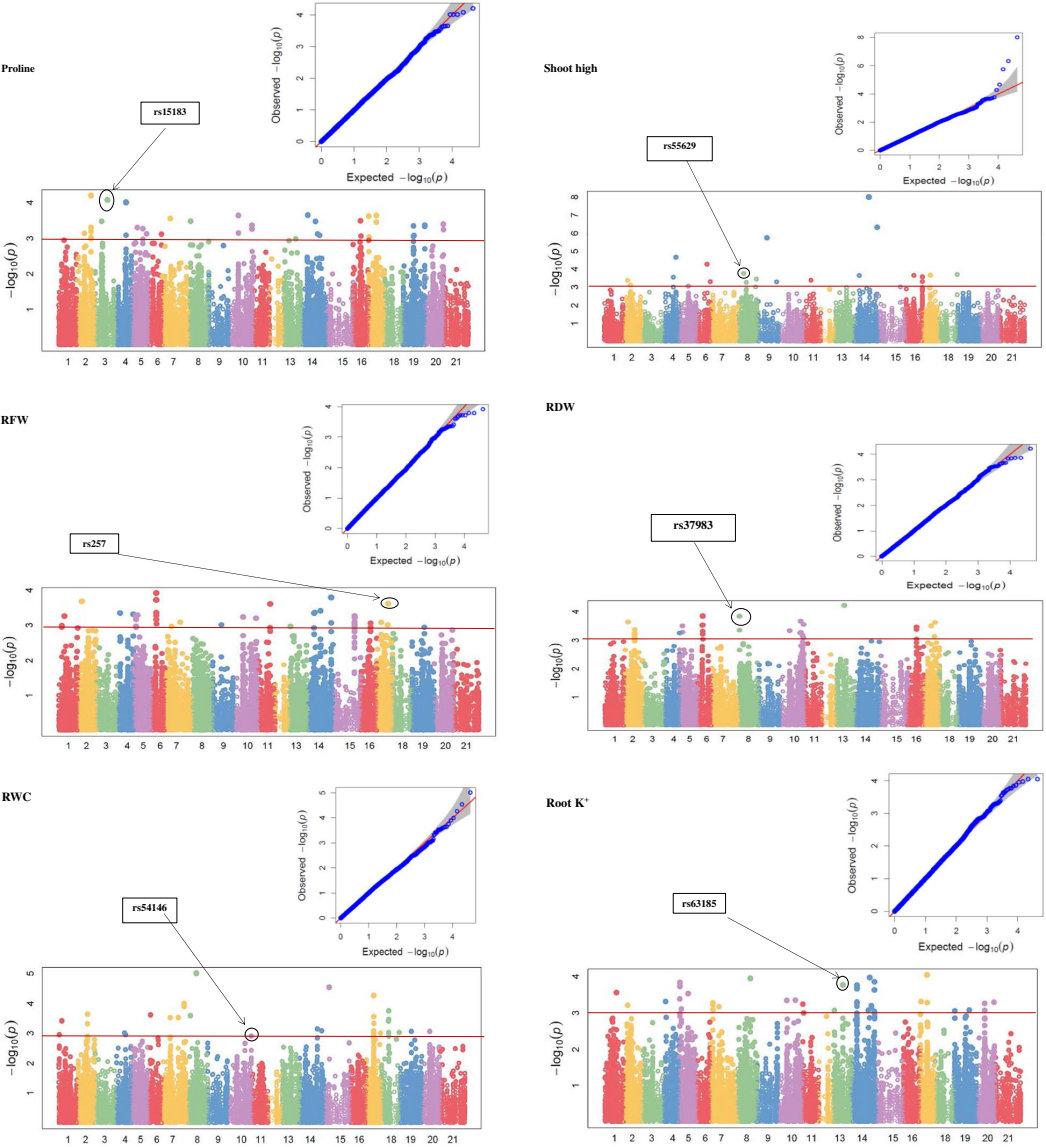
The mrMLM-based Manhattan (bottom) and QQ-plots (above) of major haplotypes for seedling-related traits under salinity conditions. X axis represents chromosome number [1)1A, 2)1B, 3)1D, 4)2A, 5)2B, 6)2D, 7)3A, 8)3B, 9)3D, 10)4A, 11)4B, 12)4D, 13)5A, 14)5B, 15)5D, 16)6A, 17)6B, 18)6D, 19)7A, 20)7B, and 21)7D] and Y axis represents –log_10_(p). The −log10 (*P*-value) ≥ 3.0 (*P* ≤ 0.001) was regarded as the significance threshold. Electrolyte leakage (ELI); SPAD; shoot fresh weight (SFW); shoot dry weight (SDW); relative water content (RWC); root fresh weight (RFW); root dry weight (RDW); root volume (RV); shoot height (SH); root height (RH); chlorophyll a (Chl a); chlorophyll b (Chl b); total chlorophyll (total Chl); carotenoid (Car); protein; proline; catalase (CAT); guaiacol peroxidase (GPX); malondialdehyde (MDA); Shoot Na (Na-s); Root Na (Na-r); Shoot K (K-s); Root K (K-r); Shoot K/Na (K/Na-s); root K/Na (K/Na-r)

Using mrMLM model, 817 and 1006 significant MTAs were identified under normal and stress conditions, respectively, for morphological, physiological, and biochemical traits at -log_10_ (*P*) > 3 (Fig. 4). Among these, 40 and 29 highly significant, functional MTAs were regarded as “reliable” MTAs under normal and stress conditions, respectively. The reliable MTAs were selected based on the fact that they passed a high significance threshold and also have a cellular function. From the reliable MTAs, we selected “major” MTAs, which explained ≥10% of the phenotypic diversity for the traits. A total of 15 and 8 major MTAs were detected for control and salt stress, respectively (Tables 5 and 6). QQ and Manhattan plots of top SNPs for the traits of interest are presented in Fig. 5.

**Table 5:** Annotation of genes harbouring the significant trait-associated SNPs across all chromosomes in Iranian wheat accessions exposed to normal conditions

Marker	Sequence	Trait	Ch.	Position (bp)	MAF^a^	R^**2**^ (%)	Gene ID in wheat	Molecular function	Biological process
rs23576	TGCAGCCCCCTCAAAGTCCAACAAAGGAAGCCTGTGTTCAAACATATCATCAGTCTTCACCCGA	ELI	7A	118,145,757-118,159,311	0.09	5.42	*TraesCS7A02G161500*	protein binding	
rs2956	TGCAGAATTCCCATATCTACCACCTGCCAAAAATTCAGCAATATCCGACCGTCAAAACTCCGAG	ELI	6B	689,197,969-689,222,878	0.20	4.94	*TraesCS6B02G417300*		
rs3669	TGCAGACCAAGTCCCTGACCGATTTAATCATTTGAACAAGTTCCTCCCGATTCAGTTAGTCAGG	SPAD	4B	389,114,118-389,118,175	0.47	10.88	*TraesCS4B02G178000*	channel activity	transmembrane transport
rs17742	TGCAGCAGGAGCTTAACGGGCCCGATCTGGGCCCGAGATCGGAAGAGCGGGATCACCGACTGCC	SFW	6D	384,280,526-384,287,890	0.12	8.25	*TraesCS6D02G275200*	hydrolase activity, acting on ester bonds D-aminoacyl-tRNA deacylase activity	D-amino acid catabolic process
rs9473	TGCAGATGGCGGCCTTCACAGAGGAGAGGAGTGAGGACACGATGGAGGAGGAGCCGTCGGCCGC	SDW	5B	571,834,531-571,837,033	0.35	10.67	*TraesCS5B02G394000*	hydrolase activity, hydrolyzing O-glycosyl compounds	carbohydrate metabolic process
rs10128	TGCAGATTCTACGCCGCTGCCTTGCCCATACTGTTATTAAGATTTAGCTCCCGCCTCGTTGCCT	RWC	1B	19,047,105-19,051,706	0.11	8.97	*TraesCS1B02G039600*	protein kinase activity, protein binding, ATP binding	protein phosphorylation
rs2460	TGCAGAATACAAGAAAACTTGGGTTGGACAGAATGCCCTTCCAACACCTCCAGGTCGAAGTTCC	RFW	2B	777,935,599-777,935,901	0.06	19.32	*TraesCS2B02G593100*	monooxygenase activity, iron ion binding, oxidoreductase activity, acting on paired donors with incorporation or reduction of molecular oxygen heme binding	
rs56706	TGCAGTAGATCAGGTGCTTGTAGCTTGACTGAACGCAATTGAAGTCTTTCCTCATAGTCGGGCT	RFW	2B	653,745,832-653,748,302	0.41	19.26	*TraesCS2B02G459300*	alternative oxidase activity	
rs43005	TGCAGGAATGCTTAGGAGTCCTGGATTACGGGGTTCTCGGGGAGCTGCCCTATGTGTCATGGGC	RDW	6B	22,081,260-22,105,393	0.09	12.94	*TraesCS6B02G037600*	protein kinase activity, calcium ion binding, ATP binding, polysaccharide binding	protein phosphorylation
rs23154	TGCAGCCCAGGGCATAGGACAGAGGCACCAAGGACCTGGCGAGATGGTGTGCACGAGGCGGGTC	RV	1B	250,982,539-251,056,868	0.39	10.15	*TraesCS1B02G153500*	oligopeptide transmembrane, transporter activity	transmembrane transport
rs2275	TGCAGAAGGTCTGAATTTGGGTGGCGTGTATGCAGGTACTCGTGCGTACACCTCCACACATGCT	RV	3A	369,616,744-369,620,089	0.39	10.06	*TraesCS5B02G203300*	protein binding	cell redox homeostasis
rs61560	TGCAGTGGCTGGACGACAAGCTCACCTCGCTCGCCCTCCCCGAACCCGAGATCGGAAGAGCGGG	SH	1B	531,203,778-531,206,255	0.07	22.4	*TraesCS1B02G308700*	protein binding, zinc ion binding	
rs61560	TGCAGTGGCTGGACGACAAGCTCACCTCGCTCGCCCTCCCCGAACCCGAGATCGGAAGAGCGGG	SH	3D	531,365,324-531,367,807	0.07	22.4	*TraesCS3D02G418800*	protein binding, zinc ion binding	
rs61560	TGCAGTGGCTGGACGACAAGCTCACCTCGCTCGCCCTCCCCGAACCCGAGATCGGAAGAGCGGG	SH	3A	665,964,671-665,967,157	0.07	22.4	*TraesCS3A02G423500*	protein binding, zinc ion binding	
rs61560	TGCAGTGGCTGGACGACAAGCTCACCTCGCTCGCCCTCCCCGAACCCGAGATCGGAAGAGCGGG	SH	1A	493,821,133-493,823,736	0.07	22.4	*TraesCS1A02G299000*	protein binding, zinc ion binding	
rs61560	TGCAGTGGCTGGACGACAAGCTCACCTCGCTCGCCCTCCCCGAACCCGAGATCGGAAGAGCGGG	SH	3B	702,690,096-702,692,579	0.07	22.4	*TraesCS3B02G459000*	protein binding, zinc ion binding	
rs61560	TGCAGTGGCTGGACGACAAGCTCACCTCGCTCGCCCTCCCCGAACCCGAGATCGGAAGAGCGGG	SH	1D	391,317,043-391,325,294	0.07	22.4	*TraesCS1D02G292300*	protein binding, zinc ion binding	
rs46112	TGCAGGCACGGCGACTGCGGGCAGCCAAGTTTTTAGTCCCACCTCGCCCGACAGAGCGCGCGAC	RH	6D	85,504,595-85,544,103	0.33	8.06	*TraesCS6D02G120400*	protein binding	
rs27832	TGCAGCGAAACCATACGATGGATGAAAATAGTACATGATGTATCAAATGGAAACTATGCCACGA	Chl a	4A	232,506,122-232,523,385	0.31	6.15	*TraesCS4A02G143000*	nucleotide binding, iron ion binding, ATP binding, ATPase, ribosomal small subunit binding, metal ion binding, iron-sulfur cluster binding	ribosomal subunit export from nucleus, translational initiation, translational termination
rs31586	TGCAGCGGATTTTTAGTCCCACCTCGCTCCGCTAACAGAGTTTTACCACATTAAATATGTTACT	Chl a	2B	613,205,484-613,234,917	0.09	6.11	*TraesCS2B02G426600*	serine-type endopeptidase activity, serine-type peptidase activity	proteolysis
rs1928	TGCAGAAGCAGCGGCACCGATAACTTCCTCCATGGGCACGATGTAAGCGGCGGTGCAGAAAGGA	Chl a	1D	317,426,727-317,457,933	0.12	5.98	*TraesCS1D02G229300*	protein serine/threonine kinase activity, protein binding, kinase activity, protein-containing, complex binding	
rs1928	TGCAGAAGCAGCGGCACCGATAACTTCCTCCATGGGCACGATGTAAGCGGCGGTGCAGAAAGGA	Chl a	1B	429,583,237-429,614,696	0.12	5.98	*TraesCS1B02G241900*	protein serine/threonine kinase activity, protein binding, kinase activity, protein-containing, complex binding	
rs40144	TGCAGCTGTACGTGCCTCCACATGTACATGTACCTCTGCCGAGATCGGAAGAGCGGGATCACCG	Chl a	2D	644,092,500-644,096,696	0.17	5.93	*TraesCS2D02G584900*	protein binding	
rs33651	TGCAGCGTGAGCAGGTTGAACAAGGGACAGACAGACAGACATGGCTGCTACACTTACCAAGTGC	Chl a	5B	707,439,895-707,441,048	0.14	5.72	*TraesCS5B02G561300*	hydrolase activity	cytokinin biosynthetic process
rs24738	TGCAGCCGACCGATAGAATTGATCCAGCCATCACTCTAGGCAGCAAGGTTCTACATCTGTGTGC	Chl b	3A	507,757,264-507,766,229	0.18	6.03	*TraesCS3A02G278200*	cysteine-type peptidase activity	proteolysis
rs30395	TGCAGCGCCTCCACCACCGACCATGATCTGCGAGGGAGCGTCTTGCTGGTGCTCATTCGATATC	Chl b	5B	703,644,434-703,646,112	0.18	5.59	*TraesCS5B02G553700*	protein binding	
rs30395	TGCAGCGCCTCCACCACCGACCATGATCTGCGAGGGAGCGTCTTGCTGGTGCTCATTCGATATC	Chl b	3B	705,135,930-705,140,149	0.18	5.59	*TraesCS3B02G462000*	metal ion binding	primary metabolic process cellular macromolecule metabolic process
rs59549	TGCAGTCTGAGAACCTTGAGGACCAGTTGACTGGTTAGGTACTGCCACTTGGCTTCTCATTTGA	Total Chl	6A	6,727,564-6,735,295	0.16	5.74	*TraesCS6A02G013700*	protein binding	
rs53598	TGCAGGTCTGGTGAGTTTGTGCTGGTCATCAGTCATCGCTCGTGCAGACGATACGAGGCTCCTA	Car	1B	677,527,503-677,530,414	0.49	7.23	*TraesCS1B02G467800*	protein kinase activity, protein binding, ATP binding	protein phosphorylation
rs59088	TGCAGTCGGAGCATCCGATGAAAATCAAATAAATTTGTTTTAGCTTCATACATACTCCAAGCAA	protein	7A	679,052,946-679,060,925	0.15	5.21	*TraesCS7A02G488800*	protein kinase activity, ATP binding	protein phosphorylation
rs14676	TGCAGCACCTTCCGCCCAATCGCCACCGACTGCTCCTTCCGCCGCCGATTCCGCCGAGATCGGA	proline	7D	607,607,584-607,615,127	0.17	32.55	*TraesCS7D02G502100*	protein binding	
rs3861	TGCAGACCCCTTTCCAGAACAGCCTCCGCGAGGTGCTGGAGGATGAGGAGGGGGTGCCGAGATC	CAT	3A	728,320,620-728,322,996	0.08	6.32	*TraesCS3A02G507200*	UDP-glycosyltransferase activity, hexosyltransferase activity	
rs9866	TGCAGATTACATCAAGGAGGACACCCCCGCCGACGGGCTCGGTGATCTGCCCGCCCAGCCACCG	GPX	1B	313,080,958-313,101,223	0.41	5.85	*TraesCS1B02G174400*	zinc ion binding	
rs9047	TGCAGATGAGGCGGTGGACGATGCGGTCGATGCAGTCCTGGGCGTCGTGCACCAGGCCAAGCAT	MDA	5A	548,503,107-548,507,985	0.07	6.37	*TraesCS5A02G344000*	ADP binding	
rs63113	TGCAGTTCCAAATTGCCCATAACAACGCATACACTCCTACACGAATATGTCTAGCTGTATCGGA	Na-s	5B	108,985,650-108,994,401	0.08	5.37	*TraesCS5B02G086000*	serine-type carboxypeptidase activity	proteolysis
rs58688	TGCAGTCCGTTTTTAATTTCTGGCCTGGATCAGTTTCTTCCTCTGGATGGCCACGCTTATTTGT	Na-r	2B	692,871,667-692,873,184	0.16	10.13	*TraesCS2B02G495900*	protein binding	
rs60006	TGCAGTGACCACGAGGCGGGGTTTCGGTCGGACGACGACTTCACGTGTCCGAGATCGGAAGAGC	K-s	4D	505,330,644-505,333,517	0.06	7.61	*TraesCS4D02G356300*	oxidoreductase activity, oxidoreductase activity, acting on the aldehyde or oxo group of donors, NAD or NADP as acceptor, fatty-acyl-CoA reductase activity, alcohol-forming fatty acyl-CoA reductase activity	lipid metabolic process
rs10679	TGCAGCAAAAATGCACGCACTCATCAGTGCTGGGTTGTGTTTCATGGGTTTCTTTACCTTTCTT	K-r	3B	75,972,378-75,978,353	0.07	9.49	*TraesCS3B02G110100*	transferase activity, glycosyltransferase activity, cellulose synthase (UDP-forming) activity, mannan synthase activity	plant-type primary cell wall biogenesis cellulose biosynthetic process cell wall organization plant-type cell wall organization or biogenesis mannosylation
rs37461	TGCAGCTCGGCCAGCTCCGCGAGCAGCGCCGCGTCGGCCGACGACTTGGACATGTCGCCGAGAT	K/Na-s	1B	411,987,863-411,990,884	0.28	5.62	*TraesCS1B02G229400*	protein kinase activity, protein serine/threonine kinase activity, ATP binding	protein phosphorylation
rs17596	TGCAGCAGCTTCTCGAATACATGGCTAGAGGACGCCACCAAACTGATGAGCTCTGCTGTGAGTG	K/Na-r	6A	430,341,106-430,343,509	0.11	7.91	*TraesCS6A02G228200*	hydrolase activity, acting on ester bonds	

^a^ *MAF* Minor allele frequency

**Table 6:** Annotation of genes harbouring the significant trait-associated SNPs across all chromosomes in Iranian wheat accessions exposed to salinity stress

Marker	Sequence	Trait	Ch.	Position (bp)	MAF	R^**2**^ (%)	Gene ID in wheat	Molecular function	Biological process
rs15925	TGCAGCAGAGAGGCGCGGAAACACGCGATCTCCGCACGCTGGGCCGCCCCAGTGGGCGGCGGTC	ELI	7A	200,599,649-200,608,278	0.25	7.27	*TraesCS7A02G230100*	antiporter activity, xenobiotic transmembrane transporter activity	transmembrane transport
rs15925	TGCAGCAGAGAGGCGCGGAAACACGCGATCTCCGCACGCTGGGCCGCCCCAGTGGGCGGCGGTC	ELI	7A	200,599,649-200,608,278	0.25	7.27	*TraesCS7A02G230100*	antiporter activity, xenobiotic transmembrane transporter activity	
rs7347	TGCAGAGTTATAGGGAAGAAGAAGAAGGCGTACGTGGAAAAAACGATTCGAGGAGCGCTCCCGT	SPAD	2A	373,418,967-373,421,254	0.31	8.43	*TraesCS2A02G249000*	electron transfer activity, protein-disulfide reductase activity, glutathione oxidoreductase activity	cell redox homeostasis
rs53540	TGCAGGTCTCGCTAATCGATCCTCGCTTTTTTTTGAGCATCAGTACAGACACAAGCGCTCATAT	SFW	6A	264,830,763-264,834,472	0.31	6.97	*TraesCS6A02G192900*	protein kinase activity ATP binding	protein phosphorylation
rs35884	TGCAGCTCACGTAGAAGGAGACCCGACCGACAGCGCGATTCGCAAGACAGTCGACGAGCGCTTT	SDW	3B	768,470,821-768,482,837	0.12	9.08	*TraesCS3B02G526100*	hydrolase activity, hydrolyzing O-glycosyl compounds	carbohydrate metabolic process
rs54146	TGCAGGTGGAAAATGGAATCGCTAGGCCGCCGCCGAGATCGGAAGAGCGGGATCACCGACTGCC	RWC	4A	685,748,621-685,752,222	0.21	10.33	*TraesCS4A02G415700*	protein binding	
rs257	TGCAGAAAAGTAAGAAATTTGAAGGAGTTTTGTTCAATCACCATTTTATTACGTGTCCTCCCGA	RFW	7B	594,048,516-594,116,949	0.10	17.48	*TraesCS7B02G339500*	protein binding	
rs37983	TGCAGCTCTGACCGACTCCGCCTGAAGCCGCCATCGTTGCCACACAGGAGGACGACCTATTATT	RDW	3B	325,816,754-325,873,702	0.18	16.57	*TraesCS3B02G227800*	protein binding	
rs18682	TGCAGCAGTGGTGGTGTGCCCTTGGTCCATGCCATGTTTGTGTGCTCACCCTGTGGTTGTGGTG	RV	1B	688,351,698-688,354,737	0.38	10.14	*TraesCS1B02G480700*	DNA binding	nucleosome assembly regulation of transcription
rs55629	TGCAGTAAACCAATCAAAATGCATGGAACTCGCAGCGCTGCTCCCGCTTGTTCCCTTCGCCG	SH	3B	193,605,454-193,613,941	0.43	22.64	*TraesCS3B02G182700*	endoribonuclease activity, producing 5′-phosphomonoesters	tRNA processing, tRNA 3′-trailer cleavage
rs44076	TGCAGGAGATGGAGGGGAGCAGTAGGGGGGTTCTCTGCTCCGCAATCAGGGATCCGAGATCGGA	RH	2D	417,090,322-417,092,425	0.31	5.79	*TraesCS2D02G324100*	protein binding	
rs34693	TGCAGCTACGGCGACGGCGGATGGGGCCTTGTTGGTCACCCCACTGCGCGTCGCAGCGCCTAGG	Chl a	7B	524,991,414-525,003,565	0.18	7.05	*TraesCS7B02G289500*	DNA binding, DNA clamp loader activity, ATP binding, ATPase	resolution of meiotic recombination intermediates, DNA replication and repair, response to abscisic acid, regulation of chromatin silencing, regulation of histone H3-K9 methylation
rs18445	TGCAGCAGTCAGTTTCCTCCTCCTCGACTCCGACCGCCTTCGTCACCCGAGGCGTCTCTGCGTC	Chl b	6A	580,203,253-580,213,091	0.29	6.21	*TraesCS6A02G347900*	ionotropic glutamate receptor activity, ligand-gated ion channel activity	
rs34693	TGCAGCTACGGCGACGGCGGATGGGGCCTTGTTGGTCACCCCACTGCGCGTCGCAGCGCCTAGG	Total Chl	7B	524,991,414-525,003,565	0.18	7.48	*TraesCS7B02G289500*	DNA binding, DNA clamp loader activity, ATP binding, ATPase	resolution of meiotic recombination intermediates, DNA replication and repair, response to abscisic acid, regulation of chromatin silencing, regulation of histone H3-K9 methylation
rs59624	TGCAGTCTGGCTGCGATGGTTTCCTCGCTTCCTCCACCTTCTTTAGAAAATAGAGACGGAGGCA	Car	6B	604,212,469-604,220,915	0.31	6.07	*TraesCS6B02G343300*	catalytic activity, metal ion binding	proteolysis
rs18946	TGCAGCATAGGAAACAGAGAACAAGTTAAGGCTGGTTTTAATGGTGAGTATCATATACTATTAT	protein	3B	521,699,476-521,702,489	0.29	6.2	*TraesCS3B02G322400*	proton transmembrane transporter activity	ion transport, ATP synthesis coupled proton transport
rs15183	TGCAGCACGGCTCAATCTCCTCCTGGGACAAGATGCGCGACCGTGTTGTCGCCAACTTCTAGGG	proline	1D	220,060,401-220,078,971	0.11	23.21	*TraesCS1D02G156100*	hydrolase activity, metal ion binding, metalloaminopeptidase activity	
rs10254	TGCAGATTGCGCCGCTGGGCGTGCCACACGTGGCGCGCGGCTGCTACGAGAAGGCGACGGCCAT	CAT	3B	790,521,121-790,523,205	0.16	5.1	*TraesCS3B02G556500*	transferase activity, glycosyltransferase activity, pentosyltransferase activity	
rs61179	TGCAGTGGAAGCGGATGGTTGAGGACCTGCTGGCGCTGGGCAAACTCAACAACTGCCTCGCCGT	GPX	1B	28,373,087-28,375,944	0.14	6.23	*TraesCS1B02G048300*	catalytic activity, ammonia-lyase activity, phenylalanine ammonia-lyase activity	L-phenylalanine catabolic process, cinnamic acid biosynthetic process
rs10192	TGCAGATTGAACCCATCCTATTCTTCTGATTGAATTCATCAGTTAATTAGAAGAAGGGAAATGG	MDA	Un	75,218,183-75,222,714	0.06	7.88	*TraesCSU02G083400*	ADP binding	
rs9791	TGCAGATGTTAGAAAACAGCCCTATACTCAATCAAGATGGCCTCAAATCAAAAAGTGTTCAGCA	MDA	6A	263,871,061-263,911,967	0.14	7.45	*TraesCS6A02G192800*	GTPase activity GTP binding	
rs61025	TGCAGTGCTAGCTGCATGCACGGGGGAGGCGATGCCATGGCATGGCGCGGCACGGGCACGGGCA	Na-s	1B	680,162,318-680,165,052	0.08	8.58	*TraesCS1B02G472200*	oxidoreductase activity, acting on single donors with incorporation of molecular oxygen, incorporation of two atoms of oxygen	
rs3228	TGCAGACACAAACGTCTCGTACCAGTGGAATGTGTAAAGAATAGTTGTTATATATCTTGCCATC	Na-r	4B	621,667,176-621,669,677	0.14	7.26	*TraesCS4B02G330600*	acyltransferase activity, transferring groups other than amino-acyl groups	
rs63185	TGCAGTTCCATATAGCCCAAAGTAATGCGCAAATTCCTATCTGAATATGTTCGGCAATAGCTGG	K-s	Un	67,782,631-67,803,633	0.45	11.67	*TraesCSU02G075800*	calcium ion binding	photosynthesis
rs63185	TGCAGTTCCATATAGCCCAAAGTAATGCGCAAATTCCTATCTGAATATGTTCGGCAATAGCTGG	K-s	5A	204,996,408-205,013,771	0.45	11.67	*TraesCS5A02G109600*	calcium ion binding	photosynthesis
rs28569	TGCAGCGACTCCAGCGTGTCCGACTTGTCGCCGTCCGTGGCCGCCGTGGCCGCGCGCACCACCA	K-r	1A	511,009,417-511,015,060	0.44	9.92	*TraesCS1A02G320400*	potassium ion transmembrane transporter activity	potassium ion transmembrane transport
rs85	TGCAGAAAAATAAAAGTTAGTTATTCGGTTGTAACCGACATAAGCTATCTCTCCAGCACGGCAG	K/Na-s	1B	396,597,678-396,605,441	0.34	8.2	*TraesCS1B02G219500*	ubiquitin-protein transferase activity, protein binding	protein ubiquitination interstrand cross-link repair
rs30786	TGCAGCGCGGCGATGACCAGGGTGACAAGGTCTCGCGGAGGCAGCGCGAGCGGGCTCCTTCAGG	K/Na-r	Un	74,817,236-74,821,587	0.45	8.7	*TraesCSU02G082000*	sucrose synthase activity, glycosyltransferase activity	sucrose metabolic process, callose deposition in phloem sieve plate
rs30786	TGCAGCGCGGCGATGACCAGGGTGACAAGGTCTCGCGGAGGCAGCGCGAGCGGGCTCCTTCAGG	K/Na-r	6D	471,917,727-471,921,914	0.45	8.7	*TraesCS6D02G403800*	sucrose synthase activity, glycosyltransferase activity	sucrose metabolic process

### Putative candidate genes for salt tolerance

The analysis of gene ontology on 29 reliable MTAs indicated that the candidate genes harboring these SNPs encode proteins involved in several biological processes, including photosynthesis, response to abscisic acid, cell redox homeostasis, sucrose and carbohydrate metabolism, ubiquitination, transmembrane transport, and chromatin silencing under salt stress. From the homologs in rice (Tables 7 and 8), 25 putative candidate genes were detected for response to salt stress.

**Table 7:** Annotation of genes harbouring the homolog trait-associated SNPs across all chromosomes in rice under normal conditions

Marker	Sequence	Trait	Ch.	Position (bp)	***p***-value	FDR	R^**2**^ (%)	Homolog gene ID in rice	Description
rs23576	TGCAGCCCCCTCAAAGTCCAACAAAGGAAGCCTGTGTTCAAACATATCATCAGTCTTCACCCGAA	ELI	4	3,555,377-3,562,902	0.000102795	0.934867761	5.42	*OsAGO4b Os04g0151800*	Argonaute and Dicer protein, PAZ domain containing protein
rs57411	TGCAGTATCTTCGAGGGCTATGTACCTCAAGGTATCATGCAGATGGTGTCCTCTTGGAGCATCT	SPAD	1	26,923,017-26,924,486	0.00017542	0.997601354	10.88	*Os_F0640 Os01g0660700*	Protein of unknown function DUF295 family protein
rs38145	TGCAGCTCTTCAGTACTACGCACGAAGACATCTGGAAGGTGCTTTTCAAGTCCAACGAGACGTG	SFW	3	11,449,370-11,450,605	0.000138462	0.934754317	8.86	*Os03g0317900*	Similar to Eukaryotic aspartyl protease family protein
rs12892	TGCAGCAATCATATTATCCAAAGGGCTCGAAAAGTGACCCGATGGTGTTGGCACATATTGCGGC	SDW	1	2,713,300-2,717,425	0.000144389	0.991476107	10.81	*Os01g0150100*	Similar to Geranylgeranyltransferase type I beta subunit
rs19020	TGCAGCATATGTTACGACTTACGACTACAGCTATGGCGGCTTCTCAGCCTCCACCTCGCGCGAC	RWC	3	31,048,351-31,055,017	0.000167852	0.897008387	8.89	*PAP2 Os03g0753100*	MADS-box transcription factor, Inflorescence and spikelet developmen
rs2460	TGCAGAATACAAGAAAACTTGGGTTGGACAGAATGCCCTTCCAACACCTCCAGGTCGAAGTTCC	RFW	8	9,921,522-9,923,218	0.000174577	0.999283649	19.32	*OsCYP96B8 Os08g0262500*	Cytochrome P450 family protein
rs43005	TGCAGGAATGCTTAGGAGTCCTGGATTACGGGGTTCTCGGGGAGCTGCCCTATGTGTCATGGGC	RDW	2	5,131,380-5,132,629	0.000323829	0.999773413	12.93	*Os02g0192300*	Zinc finger, RING/FYVE/PHD-type domain containing protein
rs14133	TGCAGCACCAGGTTTAGTAATGGCGCGTGAAGCGCCGATTAAGCACTGCCGAGATCGGAAGAGC	RV	2	25,329,183-25,341,924	0.000155995	0.660997839	10.17	*Os02g0632500*	Arf GTPase activating protein family protein
rs20420	TGCAGCATTTTGCCACCGCGAGGGTCATAAAAGGACGATATGCCCAGAAAGAGGTGATGCACCG	SH	4	18,458,075-18,458,875	0.000100427	0.5998512	22.73	*Os04g0377932*	Similar to Gonidia forming protein GlsA
rs5991	TGCAGAGCCCACCGCTGTGGAGGCGCAACCCGAAGGCACTAGCTTGTTTGACGAGAGTGCCCGA	RH	1	3,924,401-3,926,323	0.000118389	0.410072448	8.12	*Os01g0176200*	UDP-glucuronosyl/UDP-glucosyltransferase family protein
rs27832	TGCAGCGAAACCATACGATGGATGAAAATAGTACATGATGTATCAAATGGAAACTATGCCACGA	Chl a	3	3,260,107-3,270,386	0.00012644	0.922732948	6.15	*Os03g0161100*	Similar to Viral A-type inclusion protein repeat containing protein, expressed
rs24738	TGCAGCCGACCGATAGAATTGATCCAGCCATCACTCTAGGCAGCAAGGTTCTACATCTGTGTGC	Chl b	2	35,091,247-35,099,291	0.000103314	0.945323776	6.03	*CYP97A4 Os02g0817900*	Cytochrome P450 family protein
rs7710	TGCAGATAGAACCTTGTATTTTGCTCACAAAAAAGAAGAAGATAGAACCTGGATTCTCCTTCTT	Total Chl	1	2,226,409-2,229,526	0.000111059	0.969036099	6.32	*Os01g0141300*	Similar to vacuolar sorting protein 4b
rs53598	TGCAGGTCTGGTGAGTTTGTGCTGGTCATCAGTCATCGCTCGTGCAGACGATACGAGGCTCCTA	Car	2	30,011,066-30,015,609	0.000127541	0.863340516	7.23	*Os02g0722700*	Similar to Nucleic acid binding protein
rs59088	TGCAGTCGGAGCATCCGATGAAAATCAAATAAATTTGTTTTAGCTTCATACATACTCCAAGCAA	protein	3	23,989,148-23,997,520	0.000168358	0.999760957	5.22	*Os03g0628800*	Similar to H1flk (Fragment)
rs14676	TGCAGCACCTTCCGCCCAATCGCCACCGACTGCTCCTTCCGCCGCCGATTCCGCCGAGATCGGA	proline	3	11,613,231-11,614,737	0.000173086	0.999106429	32.56	*OsFbox137 Os03g0321300*	Cyclin-like F-box domain containing protein
rs3861	TGCAGACCCCTTTCCAGAACAGCCTCCGCGAGGTGCTGGAGGATGAGGAGGGGGTGCCGAGATC	CAT	8	23,648,009-23,651,073	0.000369888	0.995744042	6.33	*CycD4 Os08g0479300*	Cyclin, A/B/D/E domain containing protein
rs9866	TGCAGATTACATCAAGGAGGACACCCCCGCCGACGGGCTCGGTGATCTGCCCGCCCAGCCACCG	GPX	10	94,937-97,746	0.000141706	0.999955014	5.85	*Os10g0101000*	Serine/threonine protein kinase domain containing protein
rs9047	TGCAGATGAGGCGGTGGACGATGCGGTCGATGCAGTCCTGGGCGTCGTGCACCAGGCCAAGCAT	MDA	5	27,441,786-27,445,901	0.000315678	0.999277164	6.37	*Os05g0551900*	Similar to EMB1865 (embryo defective 1865); RNA binding
rs63113	TGCAGTTCCAAATTGCCCATAACAACGCATACACTCCTACACGAATATGTCTAGCTGTATCGGA	Na-s	6	16,400,699-16,432,426	0.000170855	0.999948041	5.37	*OsOSC6 Os06g0483200*	Similar to cycloartenol synthase
rs58688	TGCAGTCCGTTTTTAATTTCTGGCCTGGATCAGTTTCTTCCTCTGGATGGCCACGCTTATTTGT	Na-r	12	21,230,590-21,232,506	0.000146445	0.999834851	10.13	*DHQDT/SDH Os12g0534000*	Similar to Dehydroquinate dehydratase/shikimate:NADP oxidoreductase
rs46450	TGCAGGCAGTCATGTACCAGTACTACAACTCTCGCGGCCGTGGCATCTGAGCATTGGATCACGT	K-s	6	28,699,601-28,706,417	0.000270262	0.999928028	7.98	*Os06g0688100*	Hypothetical conserved gene
rs46450	TGCAGGCAGTCATGTACCAGTACTACAACTCTCGCGGCCGTGGCATCTGAGCATTGGATCACGT	K-r	6	28,699,601-28,706,417	0.000270262	0.999928028	7.98	*Os06g0688100*	Hypothetical conserved gene
rs37461	TGCAGCTCGGCCAGCTCCGCGAGCAGCGCCGCGTCGGCCGACGACTTGGACATGTCGCCGAGAT	K/Na-s	10	22,294,896-22,297,645	0.000142161	0.566933932	5.62	*SAPK3 Os10g0564500*	Serine/threonine protein kinase, Hyperosmotic stress respons
rs774	TGCAGAAATAAATATCTTTGCCGCCCCGCATCATTGGAACCTAGTCTCAACCCGAGATCGGAAG	K/Na-r	3	34,257,858-34,263,571	0.000149427	0.999795877	7.93	*OsSCAR3 Os03g0816900*	Conserved hypothetical protein

**Table 8:** Annotation of genes harbouring the homolog trait-associated SNPs across all chromosomes in rice under salinity stress

Marker	Sequence	Trait	Ch.	Position (bp)	***p***-value	FDR	R^**2**^ (%)	Homolog gene ID in rice	Description
rs15925	TGCAGCAGAGAGGCGCGGAAACACGCGATCTCCGCACGCTGGGCCGCCCCAGTGGGCGGCGGTC	ELI	1	38,144,793-38,146,141	0.000144354	0.999599159	7.27	*Os01g0878900*	Similar to 4,5-DOPA dioxygenase extradiol-like protein
rs48518	TGCAGGCGGTTGGACATGGGCATGCCCATCGACGATTCAGACGAATACGAGATCAACAAGATAT	SPAD	11	4,522,342-4,557,911	0.00012878	0.567532444	8.44	*OsOSC7 Os11g0189600*	2,3-oxidosqualene cyclase, Triterpene synthase, Parkeol synthas
rs25433	TGCAGAGTTATAGGGAAGAAGAAGAAGGCGTACGTGGAAAAAACGATTCGAGGAGCGCTCCCGT	SFW	1	9,954,154-9,955,696	0.000150218	0.915267063	7.04	*Os01g0280200*	IQ motif, EF-hand binding site domain containing protein
rs8636	TGCAGATCGGGCTTCCCCCACTGGCTTTGCGTGCGGGCAGTTTTGGGTGGTGCTTGCTGGTGGC	SDW	12	23,805,152-23,808,859	0.000177924	0.986473673	9.28	*OsPAP1d Os12g0576600*	Metallophosphoesterase domain containing protein
rs54146	TGCAGGTGGAAAATGGAATCGCTAGGCCGCCGCCGAGATCGGAAGAGCGGGATCACCGACTGCC	RWC	2	15,310,546-15,324,161	0.000103142	0.998787304	10.33	*Os02g0458900*	Conserved hypothetical protein
rs257	TGCAGAAAAGTAAGAAATTTGAAGGAGTTTTGTTCAATCACCATTTTATTACGTGTCCTCCCGA	RFW	12	23,810,618-23,814,363	0.000259648	0.972631462	17.48	*OsPAP1c Os12g0576700*	Similar to Diphosphonucleotide phosphatase 1 precursor
rs37983	TGCAGCTCTGACCGACTCCGCCTGAAGCCGCCATCGTTGCCACACAGGAGGACGACCTATTATT	RDW	1	36,936,986-36,939,375	0.000119634	0.879468434	16.57	*Os01g0855400*	SANT domain, DNA binding domain containing protein
rs18682	TGCAGCAGTGGTGGTGTGCCCTTGGTCCATGCCATGTTTGTGTGCTCACCCTGTGGTTGTGGTG	RV	9	17,024,575-17,028,546	0.000185224	0.961706611	10.14	*OsIDI4 Os09g0453800*	1-aminocyclopropane-1-carboxylate synthase family protein
rs55629	TGCAGTAAACCAATCAAAATGCATGGAACTCGCAGCGCTGCTCCCGCTTGTTCCCTTCGCCG	SH	6	5,060,664-5,064,952	0.000155318	0.863370079	22.64	*OsGPCR Os06g0199800*	cAMP-type GPCR family protein
rs2368	TGCAGAAGTGGAGCTAGTGCAGCACGTCCTAGGTGGGTCGGCCGACTTGTCGTGCTGCTGTCCG	RH	1	689,788-693,923	0.000287879	0.948801211	5.86	*Os01g0112800*	Disease resistance protein domain containing protein
rs34693	TGCAGCTACGGCGACGGCGGATGGGGCCTTGTTGGTCACCCCACTGCGCGTCGCAGCGCCTAGG	Chl a	2	3,353,590-3,358,320	0.000242824	0.999635105	7.06	*OsENODL6 Os02g0162200*	Similar to Early salt stress and cold acclimation-induced protein 2–3
rs53998	TGCAGGTGCCTTGTTGCGTGATAGGCCGCCCCATCGGCTCCATGGGCAGCCAGCGATCCCTCCA	Chl b	10	22,124,277-22,127,759	0.000115546	0.96757668	6.42	*Os10g0561300*	Similar to Monosaccharid transporter
rs34693	TGCAGCTACGGCGACGGCGGATGGGGCCTTGTTGGTCACCCCACTGCGCGTCGCAGCGCCTAGG	Total Chl	2	3,353,590-3,358,320	0.000109509	0.997017439	7.48	*OsENODL6 Os02g0162200*	Similar to Early salt stress and cold acclimation-induced protein 2–3
rs59624	TGCAGTCTGGCTGCGATGGTTTCCTCGCTTCCTCCACCTTCTTTAGAAAATAGAGACGGAGGCA	Car	6	14,281,547-14,290,711	0.000121166	0.93879979	6.07	*OsGELP83 Os06g0351500*	Lipase, GDSL domain containing protein
rs18946	TGCAGCATAGGAAACAGAGAACAAGTTAAGGCTGGTTTTAATGGTGAGTATCATATACTATTAT	protein	4	7,136,795-7,140,421	0.000114795	0.95311657	6.2	*Os04g0206200*	DNA helicase domain containing protein
rs15183	TGCAGCACGGCTCAATCTCCTCCTGGGACAAGATGCGCGACCGTGTTGTCGCCAACTTCTAGGG	proline	7	23,965,804-23,970,059	0.000152893	0.92929063	23.21	*OsWD40–145 Os07g0588500*	WD40 repeat-like domain containing protein
rs27492	TGCAGCCTGTTCCTCAATCAGTGAAGGCGCGCTGCACTCCGAGATGATCTTCAATCTTCAAGAG	CAT	5	1,199,358-1,201,038	0.000103556	0.989061867	5.49	*Os05g0121900*	Similar to Phosphate/phosphoenolpyruvate translocator protein-like
rs61179	TGCAGTGGAAGCGGATGGTTGAGGACCTGCTGGCGCTGGGCAAACTCAACAACTGCCTCGCCGT	GPX	7	306,054-306,968	0.00017113	0.906304397	6.23	*Os07g0105600*	Photosystem II oxygen evolving complex protein PsbQ family protein
rs10192	TGCAGATTGAACCCATCCTATTCTTCTGATTGAATTCATCAGTTAATTAGAAGAAGGGAAATGG	MDA	2	34,853,787-34,855,494	0.000141883	0.854263863	7.88	*Os02g0813600*	Thiolase-like, subgroup domain containing protein
rs61025	TGCAGTGCTAGCTGCATGCACGGGGGAGGCGATGCCATGGCATGGCGCGGCACGGGCACGGGCA	Na-s	3	8,679,164-8,682,334	0.000122977	0.68045149	8.58	*Os03g0263900*	EF-HAND 2 domain containing protein
rs3228	TGCAGACACAAACGTCTCGTACCAGTGGAATGTGTAAAGAATAGTTGTTATATATCTTGCCATC	Na-r	4	28,744,397-28,747,841	0.000128791	0.980216786	7.26	*OsRFPH2–14 Os04g0571200*	Similar to OSIGBa0111L12.9 protein
rs63185	TGCAGTTCCATATAGCCCAAAGTAATGCGCAAATTCCTATCTGAATATGTTCGGCAATAGCTGG	K-s	1	41,251,235-41,272,093	0.000116307	0.562674471	11.67	*Os01g0939700*	Similar to Esterase D (EC 3.1.1.1)
rs28569	TGCAGCGACTCCAGCGTGTCCGACTTGTCGCCGTCCGTGGCCGCCGTGGCCGCGCGCACCACCA	K-r	6	5,018,088-5,020,389	0.000136869	0.46984536	9.92	*OsRLCK202 Os06g0198900*	Tyrosine protein kinase domain containing protein
rs10633	TGCAGATTTTTTGATTTCAGAAGGCACTCGACAGCGGCACCGTGGAAGTCCATCAAACTGCCGA	K/Na-s	8	19,382,952-19,386,574	0.000152288	0.959659751	8.67	*Os08g0405700*	Similar to Copper chaperone homolog CCH
rs26891	TGCAGCCTCGGCATCTCCCGTACTCGCTGCTCCCGAGATCGGAAGAGCGGGATCACCGACTGCC	K/Na-r	2	15,310,546-15,324,161	0.000100334	0.40666194	8.74	*Os02g0458900*	Conserved hypothetical protein

### Genomic prediction (GP)

**Fig. 6:**
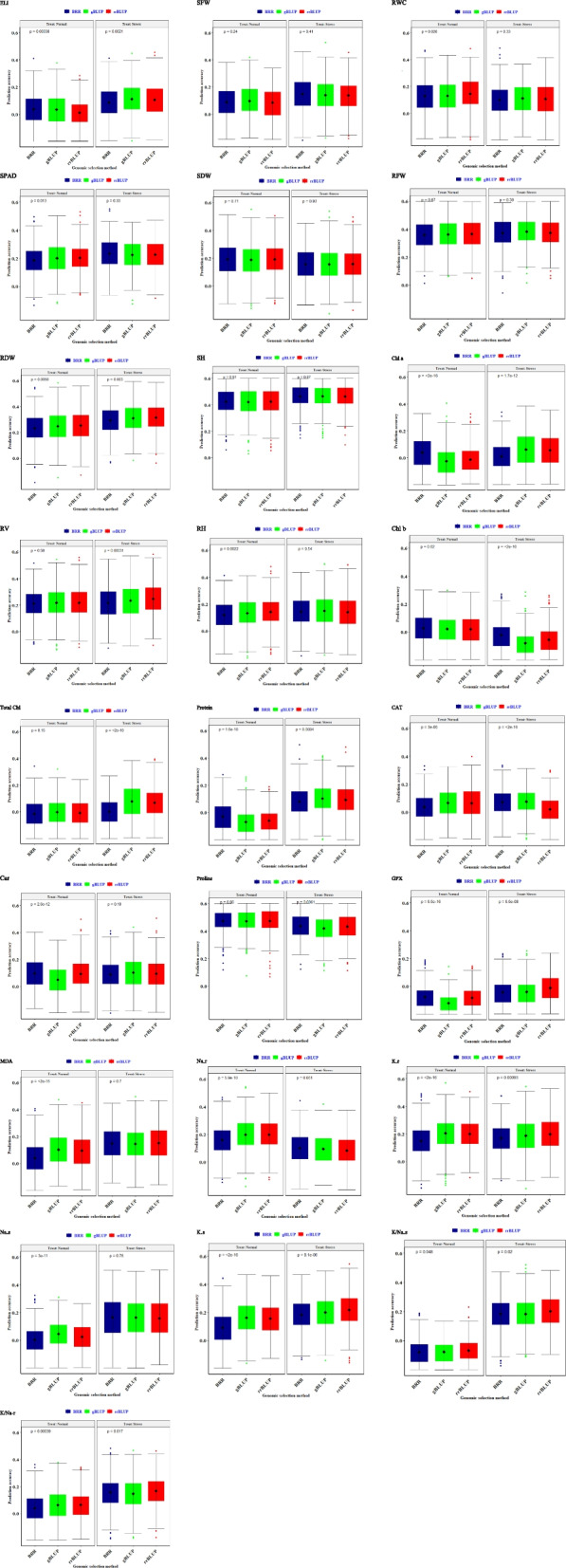
The impact of genomic selection (GS) methods on genomic prediction (GP) accuracy for 25 various traits in Iranian wheat landraces and cultivars under normal and salinity conditions. The prediction accuracy for RR-BLUP, GBLUP, and BRR-based genomic selection (GS) is presented with green, red, and blue colors, respectively. The middle point of boxplots indicates a mean of GP accuracies for the trait of interest. Electrolyte leakage (ELI); SPAD; shoot fresh weight (SFW); shoot dry weight (SDW); relative water content (RWC); root fresh weight (RFW); root dry weight (RDW); root volume (RV); shoot height (SH); root height (RH); chlorophyll a (Chl a); chlorophyll b (Chl b); total chlorophyll (total Chl); carotenoid (Car); protein; proline; catalase (CAT); guaiacol peroxidase (GPX); malondialdehyde (MDA); Shoot Na (Na-s); Root Na (Na-r); Shoot K (K-s); Root K (K-r); Shoot K/Na (K/Na-s); root K/Na (K/Na-r)

Under stress, the highest genomic prediction accuracy was achieved for RWC, ELI, chlorophyll, carotenoid, protein, and CAT traits by the GBLUP method. By the RR-BLUP method, the highest prediction accuracy was observed for GPX, root volume, and K^+^ content traits. The BRR method showed the highest prediction accuracy for SPAD and proline traits (Fig. 6). Overall, the GBLUP model exhibited better performance than BRR and RR-BLUP, suggesting that GBLUP is the preferable tool to use for genomic selection in the wheat panel.

## Discussion

Breeding for salt tolerance in wheat is a challenging task due to the polygenic nature of this trait and the polyploid nature of the wheat genome. This task is further complicated by the fact that various mechanisms are adopted for salinity tolerance at the seedling and adult growth stages [24]. To the best of our knowledge, little is known about genomic regions associated with salt tolerance at the seedling stage in wheat. With such a situation in mind, we developed a GWAS panel consisting of 298 Iranian bread wheat accessions and used this panel to identify candidate genes involved in controlling salinity tolerance at the seedling stage.

### The impact of salinity on wheat seedling traits

In-depth phenotyping is a key part of a GWAS procedure [29]. Herein, a total of 25 seedling-linked traits were evaluated that have been previously employed for QTL mapping of salinity tolerance at the seedling stage in cotton, rice, and maize [7, 9, 10]. Similar to our observations, previous reports have also shown that salinity negatively affects seedling-related traits [29–32]. In a conclusion, salt stress remarkably limits wheat seedling growth, as previously reported by Liang et al. [9].

From our findings, a negative correlation was found between Na^+^ levels and root volume, showing the detrimental effect of sodium ions on the root system. The inherent capability of accessions to maintain low Na^+^ levels is thus one of the critical parameters inducing salt tolerance. Other mechanisms for salt tolerance include tissue tolerance and Na^+^ compartmentalization which may be also involved in salinity tolerance at the seedling stage in wheat accessions [33].

### Population structure of the wheat panel

Structure analysis disclosed three subpopulations among 298 Iranian bread wheat accessions. The results from the PCA also support this observation. Interestingly, the clustered pattern of wheat accessions was not consistent with their geographical distribution or origins (Table S1, Table S2, and Fig. 3). This can be likely attributed to the migration of farmers to different regions and germplasm exchange across institutes and researchers across the world [32].

### Linkage disequilibrium in wheat sub-genomes

In line with previous reports, most markers were located in the B and A genomes [34], and the same trend was recorded for MPs in LD. The higher variation observed in the A and B genomes is likely a consequence of two factors [35], the older evolutionary history of these genomes and gene flow from the species *T. turgidum* (but not *Ae. tauschii*) to common wheat. From our observations, LD and marker distance across the A and B genomes were much lower than in the D genome. The fact that cultivars exhibit higher LD in contrast to landraces is likely a result of selection events during crop breeding [23]. In addition to selective breeding, other factors such as recombination, population relatedness, genetic drift, mutation, and mating systems affect linkage disequilibrium in wheat and other plants [36].

### Candidate genes for salt tolerance at the seedling stage

To date, many genes and QTLs connected with salinity tolerance at the seedling stage have been reported by association and linkage mapping in various crops and plants. However, little is known about the link between genomic regions associated with seedling salt tolerance with corresponding mechanisms in bread wheat. We successfully identified 27 putative candidate genes for salinity response that encode proteins/enzymes involved in antiporter, electron transfer, kinase, hydrolase, endoribonuclease, ATPase, glutamate receptor, metalloaminopeptidase, glycosyltransferase, oxidoreductase, acyltransferase, calcium ion binding, ubiquitin transferase, sucrose synthase, etc. From mapping wheat SNPs on the rice genome, 25 putative candidate genes, including *OsPAP1d*, *OsPAP1c*, *OsIDI4*, *OsGPCR*, *OsENODL6*, *OsGELP83*, *OsWD40*, *OsRFPH2*, and *OsRLCK202* were shown to be responsive to salinity. We must remind that the genomic regions associated with seedling salt tolerance, it is a problematic comparison across various studies because of the difference in the mapping population and marker platforms, as well as the absence of a consensus map for comparing genomic locations.

### Candidate genes for root/shoot height and weight

Root and shoot height and weight are key traits that specify plant architecture and affect grain yield in salt environments. The genetic basis of these traits is complex, and controlled by many genes and the environment [32]. To date, several genes have been found to be responsible for controlling root/shoot height and weight at the seedling stage of various plants [10, 28–32]. In this study, the markers rs53540, rs35884, rs257, rs37983, rs18682, rs55629, and rs44076 were linked to shoot fresh weight, shoot dry weight, root fresh weight, root dry weight, root volume, root length, and shoot height traits, respectively, allowing the identification of reliable salt-responsive genes. Among these, *TraesCS1D02G156100*, *TraesCS3B02G182700*, *TraesCS7B02G339500*, *TraesCS3B02G227800*, *TraesCS4A02G415700*, and *TraesCS1B02G480700* explained a large fraction of the phenotypic variance (≥ 10%) and classified as “major” candidate genes. Which can be targeted in future research. From mapping, the wheat SNPs on the rice genome, the root volume-connected SNP on the rice Ch.9 led to the detecting the *IDI4* gene of 1-aminocyclopropane-1-carboxylate synthases family, which have a critical function in response to hypoxic stress in crops [37].

### Candidate genes for RWC and proline content

Two major candidate genes *TraesCS1D02G156100* and *TraesCS4A02G415700* were identified that control RWC and proline and are located on Ch.1D and Ch.4A, respectively. From mapping the wheat SNPs on the rice genome, one proline-related SNP on the rice Ch.7 led to discover of a member of the WD40 protein family, *WD40–145*, which response to salt stress likely through interaction with MADS-box, MYB, and bHLH TFs [38]. Interestingly, the SPAD-connected SNP on the rice Ch.11 revealed a 2,3-oxidosqualene cyclase (OSC7), which constructs the skeleton of cyclic triterpenoids [39]. Terpenoids produced by oxidosqualene cyclases, such as α- or β-amyrin, play an essential role to cope plant roots with salinity [40].

### Candidate genes for CAT and GPX activities

In the salt-stressed seedlings, the rs10254 and rs61179 markers were detected to be associated with CAT and GPX activities, highlighting the effect of the reliable responsive genes *TraesCS3B02G556500* and *TraesCS1B02G048300*, respectively. From mapping the wheat SNPs on the rice genome, the homolog genes *Os05g0121900* and *Os07g0105600* were uncovered for affecting CAT and GPX activities on the rice Ch.5 and Ch.7, respectively. The former codes a phosphate/phosphoenolpyruvate translocator (PPT) protein-like, which is responsible for the development of phenylpropanoid metabolism-derived signal molecules triggering leaf intervene regions [41], and the latter codes a photosystem II oxygen-evolving complex protein, which is involved in transferring electrons within the cyclic electron transport pathway of photosynthesis.

### Candidate genes for pigment contents

Salt stress can inhibit PSII activity and destroy chlorophyll molecules, ultimately influencing a plant’s ability to photosynthesize [38]. To date, several QTLs for chlorophyll content has been identified during early growth stages under salinity. In our experiment, markers rs34693, rs18445, rs34693, and rs59624 were associated with to chlorophyll a, chlorophyll b, total chlorophyll, and carotenoid traits, highlighting the reliable responsive genes *TraesCS7B02G289500*, *TraesCS6A02G347900*, *TraesCS7B02G289500*, and *TraesCS6B02G343300*, respectively. Interestingly, the homolog gene *CYP97A4* was earlier identified as it influenced chlorophyll b content. Similarly, Chaurasia et al. [33] identified a gene encoding cytochrome 450, *CYP709B2*, which was involved in regulating leaf chlorophyll levels. CYPs are known to play a key role in response to salt stress by hormone signaling and/or through accelerating ROSs scavenging. Kushiro et al. [25] also uncovered an *Arabidopsis* CYP gene, *CYP709B3*, which is responsible for ABA signaling and salt response. Overall, our observation suggests that the CYP gene identified from the chlorophyll-related SNP may have a vital function in specifying wheat response to saline soils. Le et al. [43] found two SNPs for chlorophyll content located in the genes *OsRLCK253* (Ch. 8) and *OsCYL4* (Ch. 9) in salt-stressed rice. The first gene encodes a receptor-like kinase, which is known to be involved in salinity tolerance, while the second code a cyclase-containing protein, which negatively regulates stress tolerance linked to ROS levels. Le et al. [43] also detected several genes associated with chlorophyll *b* content, including *OsNUC1* (Nucleolin-like protein), *OsHox33* (HDZIP III TF), *OsARF25* (Auxin response factor), *OsWAK128* (OsWAK receptor-like kinase), *OsCHX15* (ATCHX protein), and *OsZFP213* (C2H2 TF). Moreover, we discovered one MTA for total chlorophyll content that was linked to *OsENODL6* homolog, which encodes an early nodulin-like protein in rice (located on Ch.2). Early nodulin-like proteins have been shown to display ≥3-fold changes in salt-stressed *Cajanus cajan* plants, thus, Awana et al. [42] suggested their involvement in the salt response. From mapping the wheat SNPs on the rice genome, the carotenoid-linked SNP on the rice Ch.6 uncovered *GELP83*, as a member of the GDSL esterase/lipase family, which regulates defense response, biosynthesis of secondary metabolites, and morphogenesis [44].

### Candidate genes for pigment contents

From earlier studies, genotypes tolerant to saline environments can decrease osmotic stress, absorb more K^+^, and prevent Na^+^ accumulation in order to maintain a low Na^+^/K^+^ ratio [33]. Thus, Na^+^ and K^+^-related genes were explored in our experiment to figure out K^+^ and Na^+^-dependent wheat responses to salt stress at the seeding stage.

In a high salt environment, Na^+^ toxicity and osmotic imbalance are two limiting factors for crop growth [12, 33]; so researchers have linked Na^+^ exclusion capability to grain yield under salinity stress [11]. Therefore, genes related to low Na^+^ content are key candidates for improving salt tolerance in wheat. Earlier studies have detected genomic regions associated with Na^+^ exclusion on Ch. 1A, 2A, 2B, 5B, and 6B in salt-stressed wheat [16]. Interestingly, we uncovered *TraesCS1B02G472200* and *TraesCS4B02G330600* as genes associated with Na^+^ accumulation in the shoot and root, respectively, suggesting these genes may play significant roles in sodium homeostasis at the wheat seedling stage. Chaurasia et al. [33] found three major QTNs for Na^+^ content in wheat (*Q.Na-6DL, Q.Na-6AL,* and *Q.Na-2AS*), among them, Q.Na-6DL had a remarkable contribution to Na^+^ accumulation. From mapping the wheat SNPs on the rice genome, the root Na^+^ content-related SNP on the rice Ch.4 led to the detecting of a member of RFPH protein family, *OsRFPH2–14*, which operates as RING-H2 Finger E3 ubiquitin ligase. Similarly, Liu et al. [45] reported that the *OsRFPH2–10* gene reduces the level of P2 protein and incorporates antiviral defense at the early infection stage.

In addition to Na^+^, K^+^ homeostasis is important for crop tolerance to salinity, since this ion is responsible for many key physiological processes like stomata movement, protein synthesis, respiration, photosynthesis, and growth metabolic functions [46]. In fact, higher K^+^ content may enable wheat to tolerate salt stress by developing a root system. We successfully identified *TraesCSU02G075800* and *TraesCS5A02G109600* as genes linked with K^+^ concentration in the shoot and root, respectively, suggesting these genes are important for K^+^ homeostasis at the wheat seedling stage. From the mapping of wheat SNPs on the rice genome, the root K^+^ content-related SNP on the rice Ch.6 revealed the receptor-like cytoplasmic kinase 202, *OsRLCK202*. Differential expression patterns of *OsRLCKs* at various development stages and stress suggest its involvement in diverse functions. Lin et al. [47] found a genomic region on Ch.1 associated with shoot K^+^ content (*OsHKT1*) that explained 40% of the phenotypic variation. Map-based cloning showed that this gene encodes a Na^+^ transporter, *HTK1*, which is responsible for K^+^ and Na^+^ homeostasis.

The K^+^/Na^+^ ratio is a well-known index that reflects a whole-plant response to salt stress. Generally speaking, salinity-tolerant accessions hold a low ratio of Na^+^/K^+^ in aerial parts [48]. Genomic regions related to this trait have been detected in different plants and crops and attempts are currently being made to use them in the development of high-yield cultivars tolerant to saline soils [16]. Earlier studies have reported the genomic regions on 2AL, 4AS, and 7DL associated with Na^+^/K^+^ ratio in saline fields [33, 47]. We successfully identified the genes *TraesCSU02G082000* and *TraesCS6D02G403800* for K^+^/Na^+^ ratio in shoot and root, respectively, indicating potential targets for salt tolerance breeding. Chaurasia et al. [33] reported a novel QTN (*Q.NaK-1BS)* for K^+^/Na^+^ ratio on 1BS in wheat that explain 4–38% of the phenotypic variation. Annotation of this locus demonstrated that *Q.NaK-1BS* is located inside *the Rab-like-GTPase* gene, which plays a vital function in salt tolerance by regulating Na^+^ transportation [49]. Batayeva et al. [48] found one genomic region associated with the Na^+^/K^+^ ratio on rice Ch.3 that harbored a sucrose transporter gene. Finally, Li et al. [50] discovered one novel QTL (*qSNK3–1)* located on rice Ch.3 that explains 14% of phenotypic variation. This QTL coincided with *OsIRO3* gene, which encodes a bHLH-type TF and acts as an inhibitor of Fe-deficiency response in rice.

### Genomic selection in wheat panel

The GP accuracy depends on the genomic selection method, level of LD, genetic diversity in the studied population, and genetic architecture of the studied trait [23]. In this study, we observed that the GBLUP method had better performance than the RR-BLUP and BRR methods, suggesting that GBLUP is a powerful tool for implementing genomic selection in wheat. Previous studies have suggested that high prediction accuracy can be achieved by GBLUP if markers are closely linked to the trait of interest. RR-BLUP works well for traits where the genetic architecture consists of numerous loci with small effects while the BRR approach is similar to RR-BLUP, except marker effect shrinkage depends on population size in BRR [23]. The better performance of GBLUP in our study could depend on the fact that SNPs in this study were closely associated with salt tolerance traits at the seedling stage in wheat.

## Conclusion

Our work provides new insights into the molecular mechanisms underlying salt tolerance traits at the seedling stage in wheat. Putative candidate genes controlling these traits, i.e. K^+^/Na^+^ ratio, can be targeted for developing salt-tolerant wheat cultivars at the seeding stage using marker-assisted selection. Moreover, genomic selection by using our putative genetic markers along with GBLUP-based genomic prediction will help to achieve the above-mentioned goal. Identification of varieties with high salt tolerance at the seedling stage, as well as knowledge of the associated SNPs and haplotype, could be useful for wheat production and for improvement of direct-seeding varieties.

## Material and method

### Plant material

A total of 298 Iranian bread wheat genotypes were evaluated in this study. The wheat panel contained 90 cultivars released during 1942–2014 and 208 landraces gathered from the Persian plateau during 1931–1968. All the materials were provided by the Seed and Plant Improvement Institute and the Tehran University, Karaj, Iran. More details on these bread wheat accessions can be found in Tables S1 and S2.

### Experimental design and phenotyping

The wheat cultivars and landraces were assessed for salt tolerance at the seedling stage using two salinity levels: 0 (control) and 100 (stress) mM NaCl (the selection of 100 mM NaCl stress was based on previous studies and the tolerance threshold of wheat to salinity). The study was carried out in a factorial experiment-completely randomized design (CRD) with two repeats and two factors: the first factor accounting for 298 Iranian bread wheat accessions and the second factor for two salinity concentrations. For each treatment, eight healthy and surface-sterilized seeds from each accession were planted in plastic pots (2 kg, 14 cm diameter, and 14 cm height). The soil composition of each pot was made up of a 3:2:1 ratio of decomposed litter, soil, and sand, respectively. The average temperature in the greenhouse was set to 25 °C during the day and 20 °C during the night, with a 6 h light/8 h dark photoperiod and 60% relative humidity. A thinning step was carried out at the two-leaf stage and four seedlings remained in each pot. Salt stress was gradually applied 15 days after germination by adding NaCl (25 mM) every other day together with irrigation water to reach the final concentration of NaCl, i.e., 100 mM. Crops were harvested three weeks after stress to measure the following morpho-physiological characteristics with two repeats: root volume (RV), root length (RL), shoot height (SH), root dry weight (RDW), shoot dry weight (SDW), root fresh weight (RFW), shoot fresh weight (SFW), malondialdehyde (MDA), electrolyte leakage (EL), relative water content (RWC), proline (P), soluble protein (PC), catalase (CAT), guaiacol peroxidase (GPX), photosynthetic pigments, SPAD, Na^+^ content, K^+^ content, and K^+^/Na^+^ ratio.

### Physiological trait measurements

#### Electrolyte leakage (EL)

Identical circular pieces were prepared from fully-developed leaves and placed separately in plastic-capped tubes containing distilled water for 24 h at room temperature after which the solution’s electrical conductivity (EC_1_) was measured. The tubes were put in a Ben Marie apparatus at 95 °C for 90 min, and after cooling to 25 °C, electrical conductivity (EC_2_) was measured. The EL% was calculated as (EC_1_ / EC_2_) × 100.

#### Leaf greenness

This trait was evaluated by using a SPAD-502 plus chlorophyll meter. Greenness levels were recorded based on the mean of three sections from the youngest fully-developed leaves.

#### Relative water content (RWC)

The highest leaves were harvested and their fresh weights (FW) were measured immediately. To determine the turgid weights (TW), the leaves were put down in distilled water overnight at low light intensity (to limit weight loss due to respiratory activity) and then weighted again. Eventually, leaves were placed at 70 °C for 48 h and their dry weights (DW) were recorded. Relative water content (%RWC) was estimated as: [(FW–DW)/(TW–DW)] × 100.

#### Proline content

Proline level was measured using the method developed by Bates et al. [51]. Briefly, 0.5 g of the fresh leaf was mixed with 10 mL of 3% sulfosalicylic acid and completely homogenized in a mortar. To remove excess materials from the solution, the tubes were centrifuged for 15 min at 15,000 rpm, 4 °C. The solution (2 ml) was mixed with 2 mL of ninhydrin and 2 mL of acetic acid. The tubes were kept in a hot water bath for 1 h and then cooled down in an ice bath for 1 h. Tubes containing 4 mL of toluene were vortexed for 20s and the proline content of the supernatant was estimated by a spectrophotometer at 520 nm.

#### Total protein

Leaf protein content was estimated based on Bradford [52]. Briefly, 500 mg of fresh tissue was homogenized in 5 mL of potassium phosphate buffer (10 mM, pH 7) with 5% (w/v) PVP, followed by centrifuging for 25 min at 15,000 rpm, 4 °C. Bradford reagent (990 μL) was mixed with 25 μL of supernatant and absorbance was read at 595 nm.

#### Malondialdehyde (MDA)

To detect MDA levels, as an output of lipid peroxidation, the plant extract was prepared using 1.0 g of tissue as explained by Cakmak and Horst [53]. After recording absorbance at 600 and 532 nm, the 155 mM^− 1^ cm^− 1^ extinction coefficient was used in the following formula to estimate the MDA level: nM MDA = A_532_-A _600_/1.55*10^5^.

#### Antioxidant enzyme activities

To prepare the enzymatic extract, 0.1 g of fresh tissue was crushed in liquid nitrogen, followed by adding 1 mL of sodium phosphate buffer (50 mM, pH = 7). The homogenate was centrifuged for 20 min at 10,000 rpm and 5 °C after which the CAT and GPX activities were measured from the resulting supernatant [53]. The enzyme activities were expressed as changes in absorption/min/g of fresh weight.

#### Photosynthetic pigments

Carotenoid and chlorophyll (a, b, and total) levels were measured based on the procedure described in Arnon [54]. Light absorption was read at 645 and 663 nm by a spectrophotometer and the chlorophyll levels were determined as follows:

$$\text{Chl}.\text{a}\;\left(\text{mg}/\text{g}\;\text{fresh}\;\text{weight}\right)=\left[12.7\;\left({\mathrm A}_{663}\right)-2.69\;\left({\mathrm A}_{645}\right)\right]\times\text{V}/\text{W}$$

$$\text{Chl}.\text{b}\;\left(\text{mg}/\text{g}\;\text{fresh}\;\text{weight}\right)=\left[22.9\left({\mathrm A}_{645}\right)-4.68\;\left({\mathrm A}_{663}\right)\right]\times\text{V}/\text{W}$$

$$\text{Chl}.\text{total}\;\left(\text{mg}/\text{g}\;\text{fresh}\;\text{weight}\right)=\left[20.2\;\left({\mathrm A}_{645}\right)+8.02\;\left({\mathrm A}_{663}\right)\right]\times\text{V}/\text{W}$$

Where A is the optical absorption of samples, V is the ultimate acetone volume, and W is the leaf fresh weight.

The total carotenoid was calculated as follows:

$$\textrm{Carotenoids}\ \left(\upmu \textrm{g}/\textrm{g}\right)=\frac{A\times V\times {10}^6\ }{A_{1 cm}^{1\%}\times 100\times W}$$

### K^+^/Na^+^ ratio, Na^+^ content, and K^+^ content

Three leaves of individual accessions were gathered and dried for 3 days at 55 °C and 0.5 g of dried leaves were cut into pieces and put in a digestion tube (100 ml). A total volume of 10 mL of HClO_4_ and HNO_3_ (at a 1:3 ratio) was added to the tubes. The tube was then put in a digestion block for heating for 2 days. After cooling the transparent extract, the flasks were calibrated to a final volume of 25 mL by adding distilled water. By using a Flame Photometer, the K^+^ and Na^+^ contents were estimated from the filtered solution [55].

### Phenotypic data analysis

The variance analysis (ANOVA) of data collected in the normal and salinity environments was implemented by SAS 9.4 (SAS Institute, USA). The analysis was followed by calculating Pearson’s correlation coefficient to disclose significant relationships (*P* < 0.01) between traits. The descriptive statistics of phenotypic datasets were calculated by SPSS Statistics 21.0 (IBM Inc., USA).

### Genotyping and SNP imputation

The genomic DNA was extracted from wheat seedlings by the CTAB method [56] and RNA contamination was removed using RNase. DNA concentration was checked via a Thermo Scientific NanoDrop and DNA integrity was assessed on a 0.8% agarose gel. Genotyping-by-sequencing (GBS) was done following the published protocols [57]. After constructing GBS libraries as described by Alipour et al. [58], sequencing reads were trimmed to 64 bp and grouped into sequence tags, and SNP markers were called after alignment, which permits mismatches up to 3 bp. Markers were called in TASSEL software using the UNEAK pipeline. For avoiding false positive SNPs arising from sequencing errors, SNPs were filtered out if they had a missing rate > 10%, a MAF < 1%, and heterozygosity > 10%. The remaining missing was imputed using LD KNNi in TASSEL [58]. In the SNP calling pipeline, the wheat W7984 genome assembly was regarded as the reference genome [59].

### Population structure and kinship matrix

The putative number of subpopulations (K) was determined by STRUCTURE v2.2 using 10,000 burn-in iterations, followed by 10,000 proper MCMC sample steps for K-values ranging from K = 1 to K = 10 [60]. The best-fitting K value was determined using the ΔK method [61]. The matrix of population structure (Q) was calculated for the entire sample collection using a principal component analysis (PCA) implemented with the package Tidyverse in R. The kinship matrix (K) was obtained using the package GAPIT in R [62]. For cluster analysis, the elements of the kinship matrix were regarded as similarities and the outputs were visualized using UPGMA in GAPIT [63]. A neighbor-joining tree was constructed based on a pairwise distance matrix [63] and visualized by Archaeopteryx to determine the relationship between landraces and cultivars.

### GWAS analysis

GWAS was carried out to detect marker-trait associations (MTAs) using the package mrMLM in R [21]. We considered −log_10_ (*P*-value) ≥ 3.0 (*P* ≤ 0.001) as the significance threshold based on the previous reports [58, 59]. All SNPs which met the above cut-off value were identified as significant MTAs. The GWAS results were visualized using Manhattan plots by the GAPIT package [64]. In the Manhattan plot, the x-axis and y-axis represent the chromosomal positions of SNPs and the −log_10_ (*P*-value) is derived from the F-test, respectively. Q-Q plots were also obtained to further assess the results obtained from the Manhattan plots [23].

### Candidate gene identification

To detect candidate genes affecting salinity tolerance during the seeding stage, regions surrounding traits-associated SNPs were blasted against the rice and wheat genomes in the Ensemble genome database using the BLASTn. The IWGSC RefSeq v2.0 and IRGSP 1.0 were selected as genome references for wheat and rice, respectively [59, 65]. After alignment, genes exhibiting the highest blast score and identity percentage were selected for gene ontology analyses.

### Genomic prediction (GP)

The genomic prediction was performed using three different models: Bayesian ridge regression (BRR) [66], ridge regression-best linear unbiased prediction (RR-BLUP) [67], and genomic best linear unbiased prediction (GBLUP) [68]. All GP analyses were performed using the iPat software [69]. For three subpopulations, 10, 20, and 30% of genotypes were randomly assigned to a validation set with the remaining individuals used as the training set. For all of the GP procedures, the whole prediction process was repeated 100 times for each method. The accuracy of GP was presented as Pearson’s correlation (*r*) between BLUPs and GEBVs over the training as well as validation sets.

## Supplementary Information

**Additional file 1: Table 1S.** Bread wheat cultivars used in this experiment. **Table 2S.** Bread wheat landraces used in this experiment.

**Additional file 2: Fig. 1S.** Density histogram of 25 morpho-physiological characteristics in an association panel consisting of 292 Iranian bread wheat accessions under normal and salinity conditions. Abbreviations: Electrolyte leakage (ELI); SPAD; shoot fresh weight (SFW); shoot dry weight (SDW); relative water content (RWC); root fresh weight (RFW); root dry weight (RDW); root volume (RV); shoot height (SH); root height (RH); chlorophyll a (Chl a); chlorophyll b (Chl b); total chlorophyll (total Chl); carotenoid (Car); protein; proline; catalase (CAT); guaiacol peroxidase (GPX); malondialdehyde (MDA); Shoot Na (Na-s); Root Na (Na-r); Shoot K (K-s); Root K (K-r); Shoot K/Na (K/Na-s); root K/Na (K/Na-r). **Figure 2S.** The number of subpopulations in the wheat panel based on ΔK values (a), A structure plot of 298 wheat cultivars and landraces determined by K = 3 (b).

## Data Availability

The datasets generated and analyzed during the current study are available in the Figshare repository [10.6084/m9.figshare.18774476.v1].

## Abbreviations

GP: Genomic prediction
GWAS: Genome-Wide Association Study
MTAs: Marker-trait associations
MDA: Malondialdehyde
LD: Linkage disequilibrium
QTL: Quantitative trait loci
SNP: Single nucleotide polymorphisms
RWC: Relative water content
EL: Electrolyte leakage
CAT: Catalase
GPX: Guaiacol peroxidase
EC: Electrical conductivity
GBS: Genotyping-by-sequencing
MAF: Minor allele frequencies
PCA: Principal component analysis
BRR: Bayesian ridge regression
RR-BLUP: Ridge regression-best linear unbiased prediction
GBLUP: Genomic best linear unbiased prediction
