# Supplementary material for: Genome-Wide Association Study (GWAS) and genome prediction of seedling salt tolerance in bread wheat (Triticum aestivum L.)
Source: BMC Plant Biol. 2022 Dec 13;22:581. doi: 10.1186/s12870-022-03936-8 (PMC9746167; doi:10.1186/s12870-022-03936-8)
Supplement: Supplementary file 1 — Additional file 1: Table 1S. Bread wheat cultivars used in this experiment. Table 2S. Bread wheat landraces used in this experiment. [file 12870_2022_3936_MOESM1_ESM.docx]

**Table 1S** Bread wheat cultivars used in this experiment

| Entry Number | Variety Name | Year | Synonym | Growth Habit | CIMMYT Br Line | Origin |
| --- | --- | --- | --- | --- | --- | --- |
| 1 | 4820 | 1951 |  | Spring |  | No Info |
| 2 | ADL | 1976 |  | Spring |  | Cross made in the country, one CIMMYT parent |
| 3 | AFLAK | 2010 |  | Spring | Deberia | CIMMYT advanced line |
| 4 | AKBARI | 2006 |  | Spring |  | Cross made in the country, no CIMMYT parents |
| 5 | ALBORZ | 1978 |  | Spring | SPARROW | CIMMYT segregating line or population |
| 6 | ARTA | 2006 |  | Spring |  | Cross made in the country, no CIMMYT parents |
| 7 | AZADI | 1979 |  | Facultative |  | Cross made in the country, one CIMMYT parent |
| 8 | AZAR | 1957 | AZAR, CV, AZAR, LV | Winter |  | No Info |
| 9 | BAYAT | 1976 | CHENAB 79 | Spring |  | Cross made in another country, one CIMMYT parent |
| 10 | BISTON | 1980 | BISTOON | Spring |  | Cross made in another country, one CIMMYT parent |
| 11 | CHAMRAN | 1997 |  | Spring | ATTILA | CIMMYT advanced line |
| 12 | CHAMRAN-2 | 2013 | CHAMRAN 2 | Spring |  | CIMMYT advanced line |
| 13 | DARAB 1 | 1980 |  | Spring |  | Cross made in the country, one CIMMYT parent |
| 14 | DARAB 2 | 1995 |  | Spring |  | CIMMYT advanced line |
| 15 | DASTJERDI | 1960 |  | Spring |  | No Info |
| 16 | DAYHIM | 1968 |  | Spring |  | No Info |
| 17 | DN-11 | --- |  |  |  | - |
| 18 | FALAT | 1990 | SERI-82; SERI82; SERI 82; SERI M 82 | Spring | VEERY | CIMMYT segregating line or population |
| 19 | FONG | --- |  |  |  | - |
| 20 | FONTANA | --- |  |  |  | - |
| 21 | GAHAR | 1996 | GAHER | Spring |  | CIMMYT advanced line |
| 22 | GHODS | 1988 |  | Spring |  | Cross made in the country, one CIMMYT parent |
| 23 | GOLESTAN | 1986 |  | Spring | ALONDRA'S' | CIMMYT segregating line or population |
| 24 | HOMA | 2009 |  | Winter |  | No Info |
| 25 | KARAJ 1 | 1974 |  | Facultative |  | Cross made in the country, one CIMMYT parent |
| 26 | KARAJ 2 | 1974 |  | Winter |  | No Info |
| 27 | KARAJ 3 | 1974 |  | Winter |  | No Info |
| 28 | KARIM | 2011 |  | Spring |  | No Info |
| 29 | Gascogne | 1994 |  |  |  | - |
| 30 | KAVEH | 1980 |  | Spring |  | CIMMYT segregating line or population |
| 31 | KHAZAR 1 | 1974 |  | Spring |  | CIMMYT segregating line or population |
| 32 | KOOHDASHT | 2002 |  | Spring |  | Advanced line from CIMMYT |
| 33 | MAHDAVI | 1995 |  | Spring |  | Cross made in the country, one CIMMYT parent |
| 34 | MAROON | 1991 |  | Spring |  | Cross made in the country, one CIMMYT parent |
| 35 | MIHAN | 2010 |  | Winter |  | Cross made in the country, no CIMMYT parents |
| 36 | MOGHAN 1 | 1974 |  | Spring | ANZA | Cross made in the country, one CIMMYT parent |
| 37 | MOGHAN 2 | 1974 |  | Spring |  | CIMMYT segregating line or population |
| 38 | MOGHAN 3 | 2006 | MOGHAN-3; MOGHAN3 | Spring |  | Cross made in the country, no CIMMYT parents |
| 39 | MORVARID | 2009 | MORVARIDS | Spring |  | CIMMYT advanced line |
| 40 | MV-17 | 1993 |  | Winter |  | Cross made in other country, no CIMMYT parents |
| 41 | NAVID 1990 | 1990 | NAVID | Facultative |  | CIMMYT advanced line |
| 42 | NAZ | 1978 |  | Spring | JUPATECO | CIMMYT advanced line |
| 43 | NEISHABOUR | 2006 | AKBARI | Spring |  | Cross made in the country, no CIMMYT parents |
| 44 | NICKNEJAD | 1995 |  | Spring |  | CIMMYT segregating line or population |
| 45 | OFOGH | 2012 |  | Spring |  | Cross made in the country, one CIMMYT parent |
| 46 | OHADI | 2010 |  | Winter |  | Cross made in the country, no CIMMYT parents |
| 47 | PANJAMO 62 | 1968 |  | Spring |  | - |
| 48 | PISHGAM | 2008 |  | Facultative |  | Cross made in the country, no CIMMYT parents |
| 49 | QABOOS | 2014 |  | Spring |  | - |
| 50 | RAYHANI | 1942 |  | Spring |  | No Info |
| 51 | RIJAW | 2011 |  | Facultative |  | No Info |
| 52 | SIOSSON | 1994 |  |  |  | - |
| 53 | SHAHI | 1967 |  | Winter |  | No Info |
| 54 | SHAHPASSAND | 1942 |  | Winter |  | No Info |
| 55 | SIRVAN | 2012 |  | Spring |  | Cross made in the country, two CIMMYT parents |
| 56 | SISTAN | 2006 |  | Spring |  | Cross made in the country, no CIMMYT parents |
| 57 | TAK-AB | 2013 | SOM-3 | Winter | SOM-3 CIT90038 | No Info |
| 58 | TOUS | 2002 | TOOS | Facultative | from IWWIP | CIMMYT advanced line |
| 59 | TOBARI 66 | 1969 |  | Spring |  | - |
| 60 | UROUM | 2009 |  | Winter |  | Cross made in the country, no CIMMYT parents |
| 61 | VEE/NAC | 1997 | VEERY/NAC; VEE-NAC; VEE'S'/NAC | Spring |  | No Info |
| 62 | ZARE | 2010 |  | Facultative | ESKINA | No Info |
| 63 | ZARRIN | 1995 |  | Facultative | HATUSHA | CIMMYT advanced line |
| 64 | SHANGHAI #7 | --- |  | Spring |  |  |
| 65 | INIA 66 | 1969 | INIA-66; INIA66; INIA F 66 | Spring | INIA | CIMMYT advanced line |
| 66 | ARVAND 1 | 1974 |  | Spring |  | Cross made in the country, one CIMMYT parent |
| 67 | ROSHAN | 1960 |  | Spring |  | No Info |
| 68 | RASHID | 1968 |  | Facultative |  | Cross made in the country, one CIMMYT parent |
| 69 | SABALAN | 1980 |  | Facultative |  | Cross made in the country, one CIMMYT parent |
| 70 | DARYA | 2006 | DRYA | Spring |  | Cross made in the country, no CIMMYT parents |
| 71 | ATRAK | 1995 | KAUZ | Spring | KAUZ | CIMMYT advanced line |
| 72 | BAHAR | 2007 |  | Spring |  | No Info |
| 73 | SEPAHAN | 2006 |  | Spring |  | No Info |
| 74 | BAM | 2006 |  | Spring |  | Cross made in the country, no CIMMYT parents |
| 75 | SHIRAZ | 2002 |  | Spring |  | Cross made in the country, one CIMMYT parent |
| 76 | PISHTAZ | 2002 |  | Spring |  | Cross made in the country, one CIMMYT parent |
| 77 | HAMOON | 2002 |  | Spring |  | Cross made in the country, one CIMMYT parent |
| 78 | DEZ | 2002 |  | Spring |  | CIMMYT advanced line |
| 79 | SHIROODI | 1997 |  | Spring |  | Advanced line from CIMMYT |
| 80 | MARVDASHT | 1999 |  | Spring |  | Cross made in the country, one CIMMYT parent |
| 81 | ZAGROS | 1996 |  | Spring |  | CIMMYT advanced line |
| 82 | TAJAN | 1995 |  | Spring |  | CIMMYT advanced line |
| 83 | ALVAND | 1995 |  | Facultative |  | Cross made in the country, two CIMMYT parents |
| 84 | KAVIR | 1997 |  | Spring |  | Cross made in the country, one CIMMYT parent |
| 85 | PARSI | 2009 |  | Spring |  | Cross made in the country, one CIMMYT parent |
| 86 | SIVAND | 2009 | M-84-18 | Spring |  | Cross made in the country, no CIMMYT parents |
| 87 | BEZOSTAYA | 1969 | BEZOSTAYA; BEZOSTAYA-1 | Winter |  | Cross made in other country, no CIMMYT parents |
| 88 | AKOVA | 1958 |  | Winter |  | No Info |
| 89 | SHAHRYAR | 2002 | SHAHRYAR | Winter |  | Cross made in the country, one CIMMYT parent |
| 90 | AZAR 2 | 1997 |  | Winter |  | Cross made in the country, one CIMMYT parent |

**Table 2S** Bread wheat landraces used in this experiment

| Entry Number | USDA_ PI_NO | LOC_ID | UT_ PROVINCE | UT_ CITY | UCD_ IWA_NO | PACK_ NO | UT_ ACC_NO | CIMMYT_ NO | ICARDA_ NO |
| --- | --- | --- | --- | --- | --- | --- | --- | --- | --- |
| 1 | 625281 | 164 | Gilan | GILAN | 8610616 | 4666,1 | 6779-5 | CWI72398 |  |
| 2 | 625362 | 348 | Mazandaran | BABOL | 8610990 | 5039 | 7333-4 | CWI72463 |  |
| 3 | 625433 | 247 | Khorasan | MASHHAD | 8611151 | 5201 | 6432-3 | CWI67144 | IG 122391 |
| 4 | 625661 | 247 | Khorasan | MASHHAD | 8612039 | 6090 | 1719-1 | CWI67524 |  |
| 5 | 625810 | 247 | Khorasan | MASHHAD | 8612438 | 6489 | 7156-3 | CWI72729 |  |
| 6 | 626156 | 211 | Kerman | KERMAN | 8613311 | 8274 | 5418-6 | CWI67670 |  |
| 7 | 626158 | 211 | Kerman | KERMAN | 8613313 | 8276 | 5418-8 | CWI67672 |  |
| 8 | 626215 | 211 | Kerman | KERMAN | 8613423 | 8389 | 5758-8 | CWI67751 |  |
| 9 | 626223 | 372 | Sistan-Balouchestan | ZAHEDAN | 8613434 | 8400 | 7705-2 | CWI67758 | IG 122643 |
| 10 | 626226 | 372 | Sistan-Balouchestan | ZAHEDAN | 8613437 | 8403 | 6358-2 | CWI67761 | IG 122646 |
| 11 | 626234 | 372 | Sistan-Balouchestan | ZAHEDAN | 8613445 | 8411 | 7701-3 | CWI67769 |  |
| 12 | 625080 | 308 | Markazi | SAVEH | 8609608 | 3657 | 6845-2 | CWI72118 | IG 122191 |
| 13 | 625081 | 308 | Markazi | SAVEH | 8609609 | 3658 | 6845-3 | CWI72119 | IG 122192 |
| 14 | 625123 | 327 | Markazi | KASHAN | 8609715 | 3765 | 7657-6 | CWI57793 |  |
| 15 | 625127 | 327 | Markazi | KASHAN | 8609721 | 3771 | 7655-2 | CWI72194 |  |
| 16 | 625139 | 311 | Markazi | ARAK | 8609750 | 3800 | 1761-2 | CWI72197 |  |
| 17 | 625263 | 359 | Mazandaran | MAZANDARAN | 8610561 | 4613 | 6218-2 | CWI66843 |  |
| 18 | 626260 | 372 | Sistan-Balouchestan | ZAHEDAN | 8613474 | 8441 | 7149-3 | CWI67794 |  |
| 19 | 626261 | 372 | Sistan-Balouchestan | ZAHEDAN | 8613477 | 8444 | 7694-1 | CWI67795 |  |
| 20 | 626358 | 104 | Esfahan | ESFAHAN | 8613658 | 8624 | 7518-2 | CWI67880 | IG 122754 |
| 21 | 626360 | 104 | Esfahan | ESFAHAN | 8613661 | 8627 | 7518-5 | CWI67883 | IG 122757 |
| 22 | 626565 | 104 | Esfahan | ESFAHAN | 8614310 | 9298 | 7456-1 | CWI73421 |  |
| 23 | 626566 | 104 | Esfahan | ESFAHAN | 8614311 | 9299 | 7456-2 | CWI73422 |  |
| 24 | 626573 | 104 | Esfahan | ESFAHAN | 8614330 | 9318 | 7457-2 | CWI73433 |  |
| 25 | 626699 | 203 | Ilam | ILAM | 8600080 | 281 | 5781-8 | CWI54496 |  |
| 26 | 626706 | 186 | Hamadan | MALAYER | 8600090 | 294 | 6251-2 | CWI54502 |  |
| 27 | 626736 | 255 | Khorasan | SABZVAR | 8600138 | 602 | 7003-2 | CWI70918 |  |
| 28 | 626747 | 379 | Yazd | ARDAKAN | 8600154 | 656 | 7496-2 | CWI54538 |  |
| 29 | 626764 | 384 | Yazd | YAZD | 8600186 | 758 | 7607-1 | CWI70927 |  |
| 30 | 626776 | 225 | Khorasan | BIRJAND | 8600205 | 822 | 7063-3 | CWI54569 |  |
| 31 | 626814 | 104 | Esfahan | ESFAHAN | 8600261 | 979 | 7277-2 | CWI54602 |  |
| 32 | 626825 | 143 | Esfahan | SHAHREZA | 8600276 | 1011 | 6926-5 | CWI54613 |  |
| 33 | 626846 | 384 | Yazd | YAZD | 8600308 | 1068 | 7575-3 | CWI54636 |  |
| 34 | 626855 | 344 | Markazi | VARAMIN | 8600319 | 1096 | 7034-1 | CWI54641 |  |
| 35 | 626872 | 153 | Fars | SHIRAZ | 8600342 | 1147 | 6786-2 | CWI54656 |  |
| 36 | 626908 | 216 | Kerman | SIRJAN | 8600409 | 1312 | 7272-5 | CWI54704 |  |
| 37 | 626923 | 161 | Gilan | ASTARA | 8600429 | 1342 | 6778-2 | CWI70975 |  |
| 38 | 626924 | 161 | Gilan | ASTARA | 8600430 | 1343 | 6778-3 | CWI70976 |  |
| 39 | 626932 | 200 | Hormozgan | BANDAR-ABBAS | 8600442 | 1370 | 7280-3 | CWI54727 |  |
| 40 | 626881 | 19 | Azarbayjan-Shargi | ARDABIL | 8600366 | 1181 | 3189-8 | CWI54674 |  |
| 41 | 626883 | 151 | Fars | NIRIZ | 8600368 | 1183 | 6822-2 | CWI54676 |  |
| 42 | 626895 | 37 | Azarbayjan-Shargi | MARAGHEH | 8600389 | 1267 | 3201-2 | CWI54690 |  |
| 43 | 626904 | 42 | Azarbayjan-Shargi | MIANEH | 8600405 | 1308 | 2180-2 | CWI70970 |  |
| 44 | 627072 | 398 | Zanjan | ZANJAN | 8600669 | 1698 | 6010-1 | CWI54871 |  |
| 45 | 627099 | 283 | Khouzestan | KHORAM-ABAD | 8600710 | 1759 | 6272-1 | CWI54894 |  |
| 46 | 627102 | 395 | Zanjan | ROUD-BAR | 8600715 | 1766 | 6781-1 | CWI54897 |  |
| 47 | 627103 | 357 | Mazandaran | GORGAN | 8600716 | 1768 | 7102-3 | CWI71040 |  |
| 48 | 627189 | 228 | Khorasan | BOJNOURD | 8600882 | 2009 | 7125-1 | CWI55011 |  |
| 49 | 627055 | 395 | Zanjan | ROUD-BAR | 8600643 | 1654 | 6780-1 | CWI54858 |  |
| 50 | 627057 | 162 | Gilan | FOOMAN | 8600647 | 1663 | 6835-7 | CWI71020 |  |
| 51 | 627061 | 311 | Markazi | ARAK | 8600652 | 1671 | 1756-1 | CWI71022 |  |
| 52 | 627066 | 214 | Kerman | RAFSANJAN | 8600661 | 1687 | 7262-4 | CWI54866 |  |
| 53 | 626933 | 200 | Hormozgan | BANDAR-ABBAS | 8600444 | 1373 | 7280-2 | CWI54729 |  |
| 54 | 626943 | 220 | Kerman | ZABOL | 8600461 | 1397 | 7149-2 | CWI54737 |  |
| 55 | 626958 | 14 | Azarbayjan-Gharbi | REZAIEH | 8600484 | 1430 | 6939-2 | CWI54756 |  |
| 56 | 626978 | 104 | Esfahan | ESFAHAN | 8600515 | 1472 | 7513-5 | CWI54778 |  |
| 57 | 627036 | 282 | Khouzestan | BROUJERD | 8600612 | 1619 | 6269-1 | CWI71007 |  |
| 58 | 627038 | 282 | Khouzestan | BROUJERD | 8600614 | 1621 | 6269-3 | CWI71008 |  |
| 59 | 627043 | 14 | Azarbayjan-Gharbi | REZAIEH | 8600621 | 1629 | 6347-2 | CWI54848 |  |
| 60 | 627054 | 170 | Gilan | ROUD-BAR | 8600642 | 1653 | 6779-1 | CWI54857 |  |
| 61 | 627236 | 242 | Khorasan | GONABAD | 8600968 | 2153 | 7021-1 | CWI74139 |  |
| 62 | 627299 | 381 | Yazd | DASTJERD | 8602098 | 729 | 7439-3 | CWI55130 |  |
| 63 | 627356 | 200 | Hormozgan | BANDAR-ABBAS | 8602207 | 1374 | 7280-7 | CWI55188 |  |
| 64 | 627359 | 311 | Markazi | ARAK | 8602213 | 1668 | 2031-1 | CWI55192 |  |
| 65 | 627360 | 214 | Kerman | RAFSANJAN | 8602214 | 1692 | 7262-3 | CWI55193 |  |
| 66 | 627385 | 65 | Bakhtaran | GHASRE-SHIRIN | 8602273 | 77 | 6960-12 | CWI55230 |  |
| 67 | 627399 | 394 | Zanjan | MANJIL | 8602304 | 129 | 6073-1 | CWI71169 |  |
| 68 | 627410 | 37 | Azarbayjan-Shargi | MARAGHEH | 8602331 | 158 | 6126-1 | CWI55256 |  |
| 69 | 627414 | 74 | Bakhtaran | KANGAVAR | 8602335 | 162 | 5783-1 | CWI71177 |  |
| 70 | 627416 | 91 | Bakhtaran | SANJABI | 8602337 | 164 | 6313-2 | CWI71179 |  |
| 71 | 627417 | 91 | Bakhtaran | SANJABI | 8602339 | 166 | 6469-2 | CWI55260 |  |
| 72 | 627423 | 174 | Hamadan | HAMADAN | 8602354 | 182 | 6229-3 | CWI55267 |  |
| 73 | 627460 | 255 | Khorasan | SABZVAR | 8602436 | 526 | 7002-1 | CWI71203 |  |
| 74 | 627484 | 379 | Yazd | ARDAKAN | 8602511 | 593 | 7497-4 | CWI74159 |  |
| 75 | 627787 | 52 | Azarbayjan-Shargi | TABRIZ | 8603258 | 312 | 6485-2 | CWI55542 |  |
| 76 | 627842 | 211 | Kerman | KERMAN | 8603985 | 459 | 7685-3 | CWI71344 |  |
| 77 | 627845 | 370 | Sistan-Balouchestan | ZABOL | 8603997 | 472 | 7702-2 | CWI74356 |  |
| 78 | 627849 | 370 | Sistan-Balouchestan | ZABOL | 8604008 | 484 | 7706-3 | CWI71348 |  |
| 79 | 627852 | 370 | Sistan-Balouchestan | ZABOL | 8604012 | 489 | 7708-2 | CWI71351 |  |
| 80 | 627853 | 370 | Sistan-Balouchestan | ZABOL | 8604015 | 493 | 7709-1 | CWI71352 |  |
| 81 | 627856 | 364 | Mazandaran | SARI | 8604040 | 521 | 2660-2 | CWI71356 |  |
| 82 | 627873 | 392 | Zanjan | GAZVIN | 8604084 | 571 | 5933-1 | CWI55596 |  |
| 83 | 627688 | 131 | Esfahan | NAEIN | 8603128 | 155 | 7388-5 | CWI55508 |  |
| 84 | 627723 | 384 | Yazd | YAZD | 8603178 | 219 | 2897-5 | CWI74261 |  |
| 85 | 627760 | 25 | Azarbayjan-Shargi | AZAR-SHAHR | 8603229 | 280 | 6043-3 | CWI74296 |  |
| 86 | 627551 | 19 | Azarbayjan-Shargi | ARDABIL | 8602698 | 1337 | 5620-2 | CWI74170 |  |
| 87 | 627587 | 284 | Kordestan | BANEH | 8602975 | 306 | 6100-2 | CWI74193 |  |
| 88 | 627616 | 104 | Esfahan | ESFAHAN | 8603025 | 32 | 5756-6 | CWI74214 |  |
| 89 | 627881 | 52 | Azarbayjan-Shargi | TABRIZ | 8604095 | 588 | 6482-2 | CWI71371 |  |
| 90 | 627883 | 52 | Azarbayjan-Shargi | TABRIZ | 8604098 | 591 | 6482-5 | CWI55600 |  |
| 91 | 627905 | 347 | Mazandaran | AMOL | 8604142 | 641 | 6218-1 | CWI71388 |  |
| 92 | 627908 | 311 | Markazi | ARAK | 8604146 | 645 | 6268-2 | CWI55610 |  |
| 93 | 627948 | 308 | Markazi | SAVEH | 8604263 | 790 | 6472-1 | CWI71411 |  |
| 94 | 627963 | 174 | Hamadan | HAMADAN | 8604293 | 828 | 5178-2 | CWI55642 |  |
| 95 | 627987 | 392 | Zanjan | GAZVIN | 8604358 | 908 | 5987-10 | CWI55657 |  |
| 96 | 627990 | 55 | Bakhtaran | BAKHTARAN | 8604367 | 918 | 6161-12 | CWI55659 |  |
| 97 | 628012 | 55 | Bakhtaran | BAKHTARAN | 8604459 | 1034 | 6291-1 | CWI55673 |  |
| 98 | 628084 | 357 | Mazandaran | GORGAN | 8604658 | 1288 | 6214-2 | CWI55723 |  |
| 99 | 628088 | 311 | Markazi | ARAK | 8604666 | 1299 | 6344-4 | CWI55727 |  |
| 100 | 628114 | 143 | Esfahan | SHAHREZA | 8604706 | 1345 | 7547-3 | CWI55746 |  |
| 101 | 628189 | 203 | Ilam | ILAM | 8604850 | 1510 | 6351-6 | CWI55795 |  |
| 102 | 621619 | 286 | Kordestan | BIJAR | 8609087 | 3128 | 6326-4 | CWI71852 |  |
| 103 | 621650 | 376 | Tehran | TEHRAN | 8609174 | 3215 | 497-1 | CWI74531 | IG 122108 |
| 104 | 621668 | 376 | Tehran | TEHRAN | 8609212 | 3254 | 489-8 | CWI71888 |  |
| 105 | 621669 | 376 | Tehran | TEHRAN | 8609214 | 3256 | 489-3 | CWI74548 |  |
| 106 | 621704 | 156 | Gazvin | GHAZVIN | 8609312 | 3356 | 6063-1 | CWI71907 | IG 122115 |
| 107 | 621706 | 156 | Gazvin | GHAZVIN | 8609315 | 3359 | 2754-1 | CWI71910 | IG 122118 |
| 108 | 621712 | 156 | Gazvin | GHAZVIN | 8609332 | 3380 | 5938-7 | CWI71923 |  |
| 109 | 621716 | 156 | Gazvin | GHAZVIN | 8609342 | 3390 | 5254-6 | CWI71929 |  |
| 110 | 620903 | 3 | Azarbayjan-Gharbi | AZ-GH | 8606159 | 887 | 6503-2 | CWI55925 | IG 121878 |
| 111 | 621420 | 174 | Hamadan | HAMADAN | 8607898 |  | 6301-1 | CWI57018 |  |
| 112 | 621421 | 174 | Hamadan | HAMADAN | 8607899 |  | 6301-2 | CWI57019 |  |
| 113 | 621492 | 55 | Bakhtaran | BAKHTARAN | 8608259 | 2296 | 6136-6 | CWI57240 |  |
| 114 | 621565 | 174 | Hamadan | HAMADAN | 8608669 | 2710 | 5889-3 | CWI57495 |  |
| 115 | 622084 | 359 | Mazandaran | MAZANDARAN | 8610560 | 4612 | 6218-1 | CWI72392 |  |
| 116 | 622098 | 164 | Gilan | GILAN | 8610600 | 4651 | 5778-5 | CWI66864 |  |
| 117 | 622099 | 164 | Gilan | GILAN | 8610603 | 4654 | 6070-1 | CWI66867 |  |
| 118 | 622105 | 164 | Gilan | GILAN | 8610615 | 4666 | 5747-3 | CWI72397 |  |
| 119 | 622247 | 364 | Mazandaran | SARI | 8610962 | 5011 | 5694-2 | CWI72458 |  |
| 120 | 622264 | 348 | Mazandaran | BABOL | 8610987 | 5036 | 7333-1 | CWI49895 |  |
| 121 | 622272 | 347 | Mazandaran | AMOL | 8610997 | 5046 | 7335-1 | CWI67063 |  |
| 122 | 622311 | 247 | Khorasan | MASHHAD | 8611152 | 5202 | 6432-4 | CWI72495 | IG 122392 |
| 123 | 621717 | 156 | Gazvin | GHAZVIN | 8609348 | 3396 | 6719-2 | CWI71931 |  |
| 124 | 621735 | 156 | Gazvin | GHAZVIN | 8609375 | 3423 | 5255-1 | CWI71953 |  |
| 125 | 621736 | 156 | Gazvin | GHAZVIN | 8609376 | 3424 | 5255-2 | CWI71954 |  |
| 126 | 621869 | 311 | Markazi | ARAK | 8609894 | 3943 | 6267-4 | CWI72226 |  |
| 127 | 621908 | 311 | Markazi | ARAK | 8609968 | 4017 | 2040-2 | CWI72271 |  |
| 128 | 622063 | 398 | Zanjan | ZANJAN | 8610503 | 4553 | 5934-2 | CWI72383 |  |
| 129 | 623109 | 381 | Yazd | DASTJERD | 8600170 | 723 | 7439-2 | CWI54547 |  |
| 130 | 623123 | 153 | Fars | SHIRAZ | 8600348 | 1153 | 6830-3 | CWI54661 |  |
| 131 | 623125 | 153 | Fars | SHIRAZ | 8600362 | 1178 | 6814-4 | CWI54670 |  |
| 132 | 623127 | 149 | Fars | FASA | 8600364 | 1180 | 6813-2 | CWI54672 |  |
| 133 | 623008 | 104 | Esfahan | ESFAHAN | 8614087 | 9074 | 7556-3 | CWI74680 |  |
| 134 | 623069 | 104 | Esfahan | ESFAHAN | 8614329 | 9317 | 7457-1 | CWI73432 |  |
| 135 | 623090 | 94 | Bakhtaran | SHAH-ABAD | 8600045 | 230 | 2243-1 | CWI54471 |  |
| 136 | 623091 | 247 | Khorasan | MASHHAD | 8600048 | 232 | 5806-1 | CWI70894 |  |
| 137 | 622379 | 247 | Khorasan | MASHHAD | 8611291 | 5342 | 7069-1 | CWI67210 | IG 122481 |
| 138 | 622894 | 104 | Esfahan | ESFAHAN | 8613659 | 8625 | 7518-3 | CWI67881 | IG 122755 |
| 139 | 623266 | 14 | Azarbayjan-Gharbi | REZAIEH | 8602192 | 1136 | 5208-3 | CWI55181 |  |
| 140 | 623274 | 68 | Bakhtaran | GILANE-GHARB | 8602279 | 104 | 5791-13 | CWI55234 |  |
| 141 | 623291 | 197 | Hamadan | TOYSERKAN | 8602365 | 193 | 6547-2 | CWI55276 |  |
| 142 | 623318 | 379 | Yazd | ARDAKAN | 8602543 | 631 | 7499-2 | CWI71221 |  |
| 143 | 623338 | 153 | Fars | SHIRAZ | 8602686 | 1229 | 6829-6 | CWI74167 |  |
| 144 | 623344 | 68 | Bakhtaran | GILANE-GHARB | 8602889 | 220 | 5788-3 | CWI74186 |  |
| 145 | 623345 | 284 | Kordestan | BANEH | 8602981 | 312 | 6098-1 | CWI74198 |  |
| 146 | 623377 | 217 | Kerman | SOOFI-ABAD | 8603165 | 203 | 5418-7 | CWI74252 |  |
| 147 | 623379 | 210 | Kerman | JIROFT | 8603167 | 205 | 5758-2 | CWI74254 |  |
| 148 | 623136 | 10 | Azarbayjan-Gharbi | MAHABAD | 8600439 | 1364 | 3227-6 | CWI54724 |  |
| 149 | 623139 | 153 | Fars | SHIRAZ | 8600462 | 1397 | 6823-2 | CWI54738 |  |
| 150 | 623161 | 10 | Azarbayjan-Gharbi | MAHABAD | 8600624 | 1632 | 6943-1 | CWI54849 |  |
| 151 | 623162 | 10 | Azarbayjan-Gharbi | MAHABAD | 8600626 | 1634 | 6943-3 | CWI54851 |  |
| 152 | 623169 | 170 | Gilan | ROUD-BAR | 8600668 | 1696 | 6779-5 | CWI71025 |  |
| 153 | 623176 | 225 | Khorasan | BIRJAND | 8600725 | 1779 | 7045-3 | CWI54903 |  |
| 154 | 623510 | 3 | Azarbayjan-Gharbi | AZ-GH | 8606161 | 889 | 6503-4 | CWI55927 | IG 121880 |
| 155 | 623905 | 79 | Bakhtaran | KERMANSHAH | 8607380 | 12104-1 | 5783-2 | CWI71625 |  |
| 156 | 623908 | 79 | Bakhtaran | KERMANSHAH | 8607383 | 12103 | 5783-5 | CWI56702 |  |
| 157 | 623909 | 79 | Bakhtaran | KERMANSHAH | 8607384 | 13475-7 | 5784-1 | CWI56703 |  |
| 158 | 623953 | 65 | Bakhtaran | GHASRE-SHIRIN | 8607446 | 12551-5 | 5797-5 | CWI56757 |  |
| 159 | 623980 | 197 | Hamadan | TOYSERKAN | 8607499 | 10837-4 | 5851-2 | CWI56798 |  |
| 160 | 624215 | 174 | Hamadan | HAMADAN | 8607897 |  | 6299-3 | CWI57017 |  |
| 161 | 624240 | 203 | Ilam | ILAM | 8607949 | 40733-3 | 6352-2 | CWI57057 |  |
| 162 | 624251 | 203 | Ilam | ILAM | 8607960 | 40779-8 | 6354-3 | CWI57068 |  |
| 163 | 623475 | 203 | Ilam | ILAM | 8606083 | 18704-7 | 2230-2 | CWI55893 |  |
| 164 | 623503 | 203 | Ilam | ILAM | 8606137 | 18948-5 | 2852-2 | CWI71582 |  |
| 165 | 623506 | 79 | Bakhtaran | KERMANSHAH | 8606149 | 57952-5 | 8032-1 | CWI71586 |  |
| 166 | 623507 | 79 | Bakhtaran | KERMANSHAH | 8606150 | 57957-6 | 8032-2 | CWI55919 |  |
| 167 | 623508 | 79 | Bakhtaran | KERMANSHAH | 8606151 | 57962-6 | 8032-3 | CWI55920 |  |
| 168 | 623382 | 210 | Kerman | JIROFT | 8603170 | 209 | 5758-5 | CWI74256 |  |
| 169 | 623417 | 370 | Sistan-Balouchestan | ZABOL | 8604017 | 494 | 7709-3 | CWI71353 |  |
| 170 | 623421 | 52 | Azarbayjan-Shargi | TABRIZ | 8604099 | 592 | 6482-6 | CWI71373 |  |
| 171 | 623428 | 52 | Azarbayjan-Shargi | TABRIZ | 8604394 | 950 | 6060-2 | CWI55665 |  |
| 172 | 623473 | 203 | Ilam | ILAM | 8606081 | 18937-4 | 2160-2 | CWI55891 |  |
| 173 | 624596 | 174 | Hamadan | HAMADAN | 8608622 | 2662 | 6234-1 | CWI71789 | IG 122051 |
| 174 | 624804 | 65 | Bakhtaran | GHASRE-SHIRIN | 8608963 | 3004 | 6154-1 | CWI57692 |  |
| 175 | 624805 | 65 | Bakhtaran | GHASRE-SHIRIN | 8608964 | 3005 | 6154-2 | CWI57693 |  |
| 176 | 624818 | 203 | Ilam | ILAM | 8608986 | 3027 | 382-2 | CWI71841 | IG 122095 |
| 177 | 624837 | 203 | Ilam | ILAM | 8609011 | 3052 | 6353-1 | CWI57721 | IG 122102 |
| 178 | 624838 | 203 | Ilam | ILAM | 8609012 | 3053 | 6353-2 | CWI57722 | IG 122103 |
| 179 | 624846 | 203 | Ilam | ILAM | 8609020 | 3061 | 5781-1 | CWI57730 |  |
| 180 | 624849 | 203 | Ilam | ILAM | 8609023 | 3064 | 5781-4 | CWI57733 |  |
| 181 | 624861 | 203 | Ilam | ILAM | 8609036 | 3077 | 6354-2 | CWI57745 |  |
| 182 | 624315 | 292 | Kordestan | KORDESTAN | 8608123 | 2156 | 6941-1 | CWI57161 |  |
| 183 | 624378 | 55 | Bakhtaran | BAKHTARAN | 8608261 | 2298 | 6280-2 | CWI57242 |  |
| 184 | 624381 | 55 | Bakhtaran | BAKHTARAN | 8608266 | 2303 | 6360-5 | CWI57247 |  |
| 185 | 624576 | 174 | Hamadan | HAMADAN | 8608563 | 2603 | 6222-2 | CWI57455 | IG 122021 |
| 186 | 624580 | 174 | Hamadan | HAMADAN | 8608574 | 2615 | 6147-6 | CWI57460 | IG 122029 |
| 187 | 624582 | 174 | Hamadan | HAMADAN | 8608580 | 2619 | 6229-2 | CWI57463 | IG 122032 |
| 188 | 624585 | 174 | Hamadan | HAMADAN | 8608583 | 2622 | 6242-1 | CWI71781 | IG 122035 |
| 189 | 624944 | 376 | Tehran | TEHRAN | 8609206 | 3247 | 5164-2 | CWI74544 |  |
| 190 | 624946 | 376 | Tehran | TEHRAN | 8609211 | 3253 | 5251-3 | CWI74546 |  |
| 191 | 624947 | 376 | Tehran | TEHRAN | 8609213 | 3255 | 489-2 | CWI74547 |  |
| 192 | 624956 | 376 | Tehran | TEHRAN | 8609233 | 3277 | 5261-3 | CWI71892 |  |
| 193 | 624963 | 376 | Tehran | TEHRAN | 8609251 | 3295 | 500-9 | CWI74564 |  |
| 194 | 624980 | 156 | Gazvin | GHAZVIN | 8609319 | 3363 | 6079-2 | CWI71913 | IG 122122 |
| 195 | 624983 | 156 | Gazvin | GHAZVIN | 8609322 | 3366 | 6079-6 | CWI71916 | IG 122125 |
| 196 | 624985 | 156 | Gazvin | GHAZVIN | 8609327 | 3375 | 5938-2 | CWI71918 | IG 122128 |
| 197 | 624990 | 156 | Gazvin | GHAZVIN | 8609356 | 3404 | 5239-1 | CWI71938 |  |
| 198 | 625047 | 308 | Markazi | SAVEH | 8609534 | 3582 | 6843-2 | CWI72063 | IG 122135 |
| 199 | 624863 | 203 | Ilam | ILAM | 8609038 | 3079 | 6351-1 | CWI57746 |  |
| 200 | 624864 | 203 | Ilam | ILAM | 8609039 | 3080 | 6351-2 | CWI57747 |  |
| 201 | 624894 | 286 | Kordestan | BIJAR | 8609086 | 3127 | 6326-3 | CWI74495 |  |
| 202 | 624900 | 286 | Kordestan | BIJAR | 8609094 | 3135 | 6717-5 | CWI71853 |  |
| 203 | 624901 | 286 | Kordestan | BIJAR | 8609095 | 3136 | 6717-6 | CWI74501 |  |
| 204 | 624910 | 190 | Hamadan | NAHAVAND | 8609110 | 3151 | 6282-2 | CWI74508 |  |
| 205 | 624911 | 190 | Hamadan | NAHAVAND | 8609111 | 3152 | 6282-3 | CWI74509 |  |
| 206 | 624925 | 186 | Hamadan | MALAYER | 8609153 | 3194 | 6345-1 | CWI71875 |  |
| 207 | 624939 | 376 | Tehran | TEHRAN | 8609197 | 3238 | 484-1 | CWI74537 |  |
| 208 | 624941 | 376 | Tehran | TEHRAN | 8609200 | 3241 | 484-4 | CWI74540 |  |
