# Supplementary material for: Genome-Wide Association Study (GWAS) and genome prediction of seedling salt tolerance in bread wheat (Triticum aestivum L.)
Source: BMC Plant Biol. 2022 Dec 13;22:581. doi: 10.1186/s12870-022-03936-8 (PMC9746167; doi:10.1186/s12870-022-03936-8)
Supplement: Supplementary file 2 — Additional file 2: Fig. 1S. Density histogram of 25 morpho-physiological characteristics in an association panel consisting of 292 Iranian bread wheat accessions under normal and salinity conditions. Abbreviations: Electrolyte leakage (ELI); SPAD; shoot fresh weight (SFW); shoot dry weight (SDW); relative water content (RWC); root fresh weight (RFW); root dry weight (RDW); root volume (RV); shoot height (SH); root height (RH); chlorophyll a (Chl a); chlorophyll b (Chl b); total chlorophyll (total Chl); carotenoid (Car); protein; proline; catalase (CAT); guaiacol peroxidase (GPX); malondialdehyde (MDA); Shoot Na (Na-s); Root Na (Na-r); Shoot K (K-s); Root K (K-r); Shoot K/Na (K/Na-s); root K/Na (K/Na-r). Figure 2S. The number of subpopulations in the wheat panel based on ΔK values (a), A structure plot of 298 wheat cultivars and landraces determined by K = 3 (b). [file 12870_2022_3936_MOESM2_ESM.docx]

| 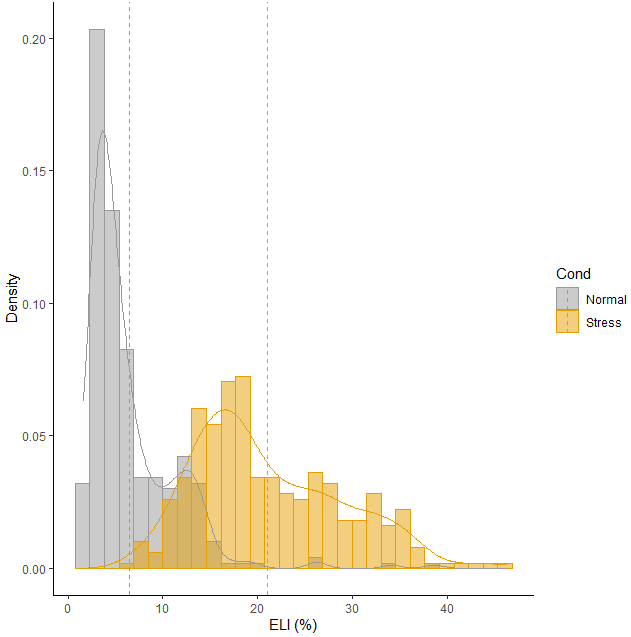 | 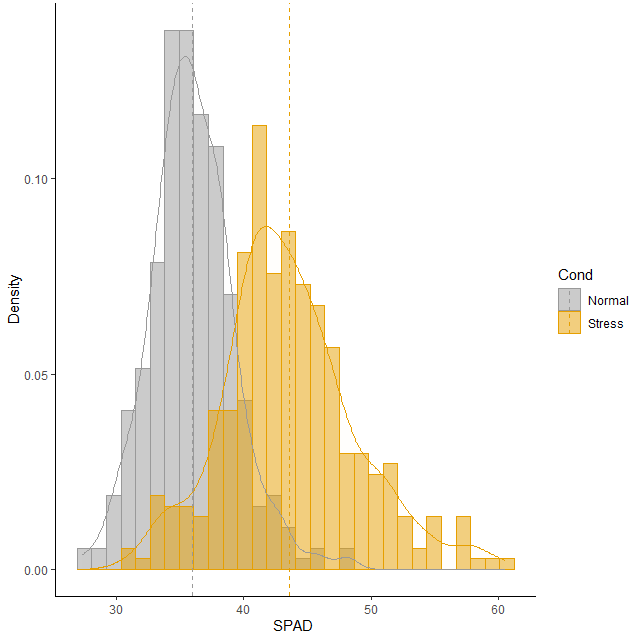 | 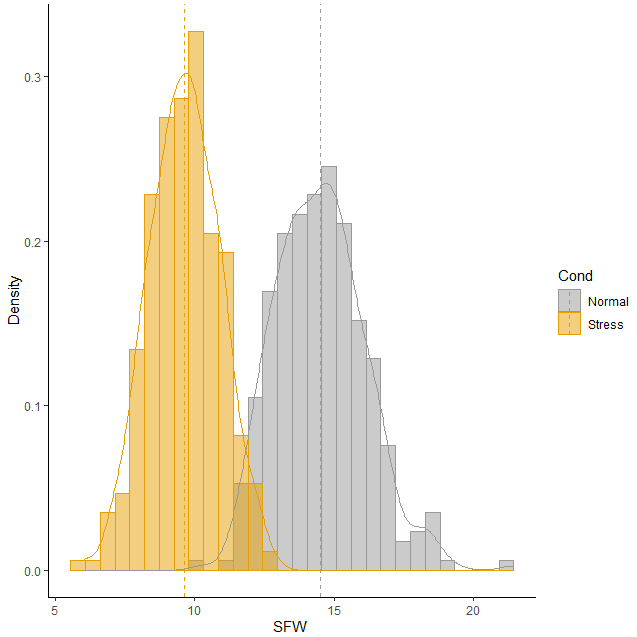 |
| --- | --- | --- |
| 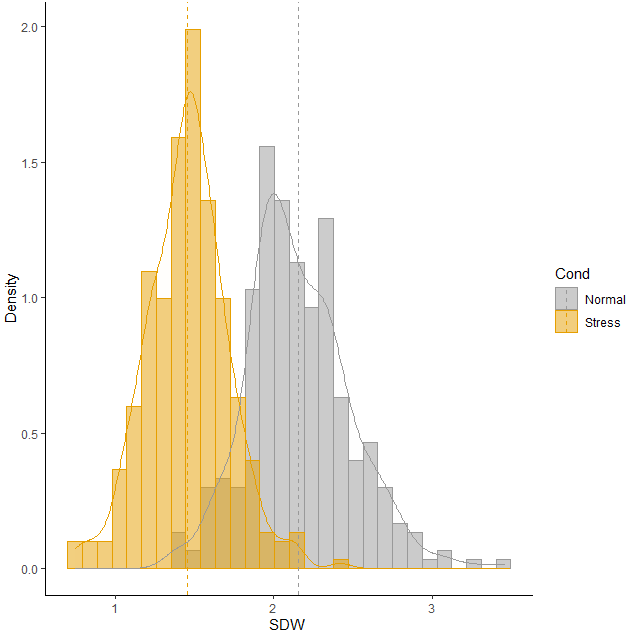 | 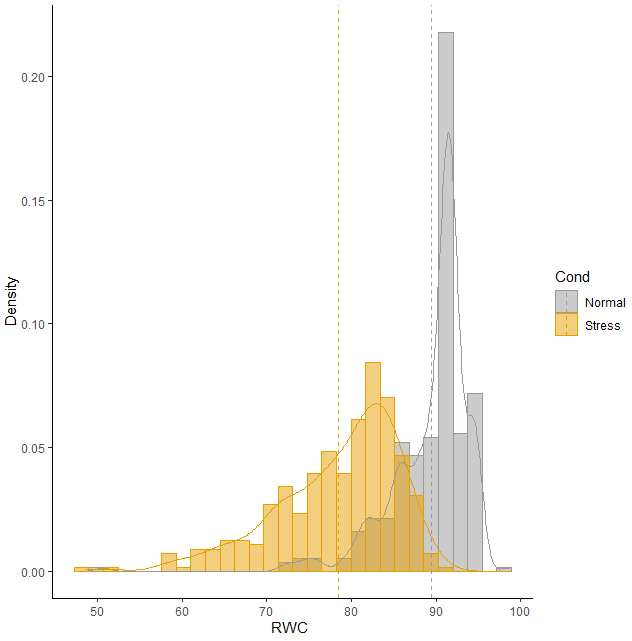 | 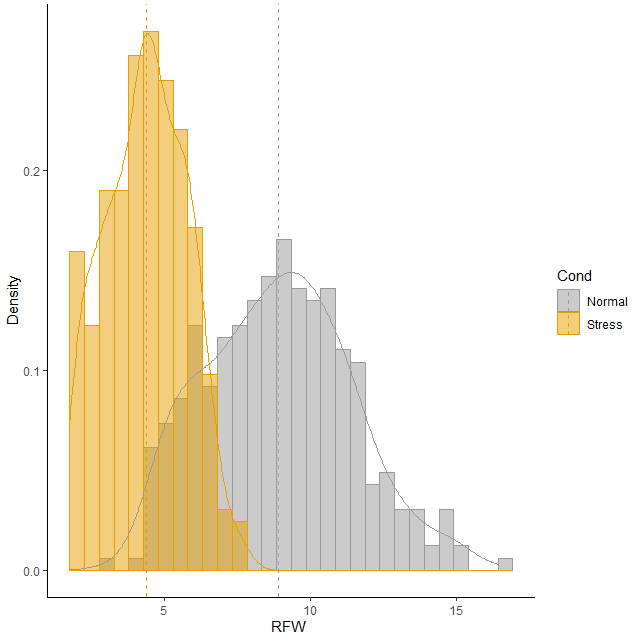 |
| 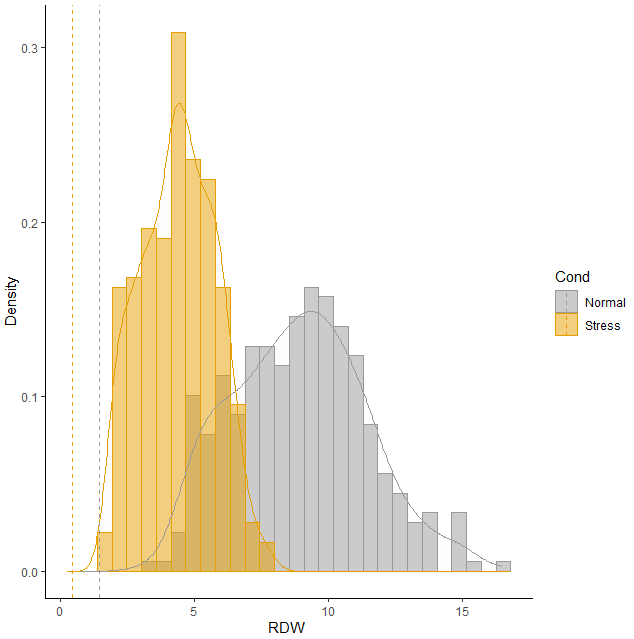 | 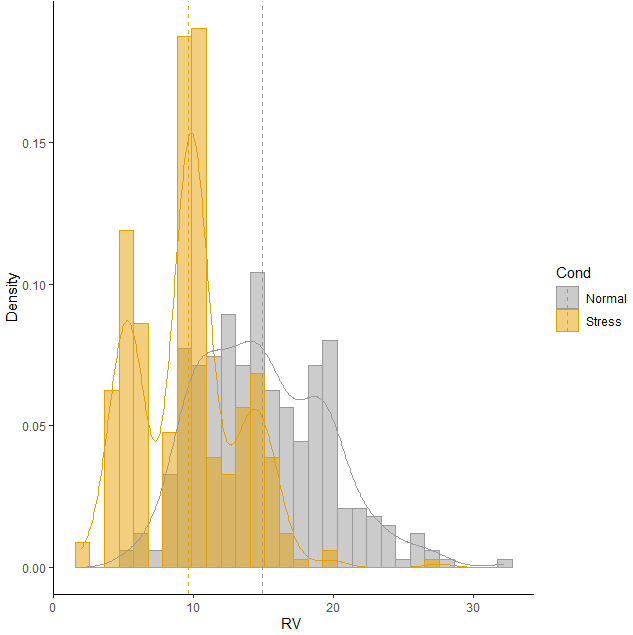 | 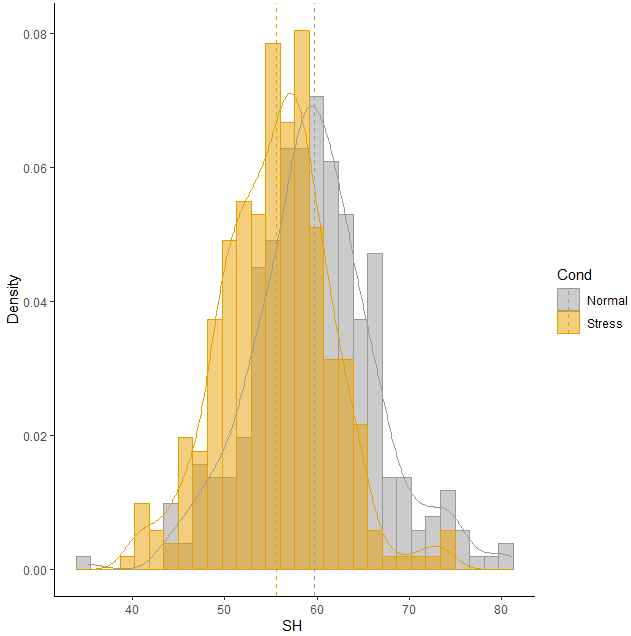 |

**Fig. 1S** Density histogram of 25 morpho-physiological characteristics in an association panel consisting of 292 Iranian bread wheat accessions under normal and salinity conditions. Abbreviations: Electrolyte leakage (ELI); SPAD; shoot fresh weight (SFW); shoot dry weight (SDW); relative water content (RWC); root fresh weight (RFW); root dry weight (RDW); root volume (RV); shoot height (SH); root height (RH); chlorophyll a (Chl a); chlorophyll b (Chl b); total chlorophyll (total Chl); carotenoid (Car); protein; proline; catalase (CAT); guaiacol peroxidase (GPX); malondialdehyde (MDA); Shoot Na (Na-s); Root Na (Na-r); Shoot K (K-s); Root K (K-r); Shoot K/Na (K/Na-s); root K/Na (K/Na-r).

| 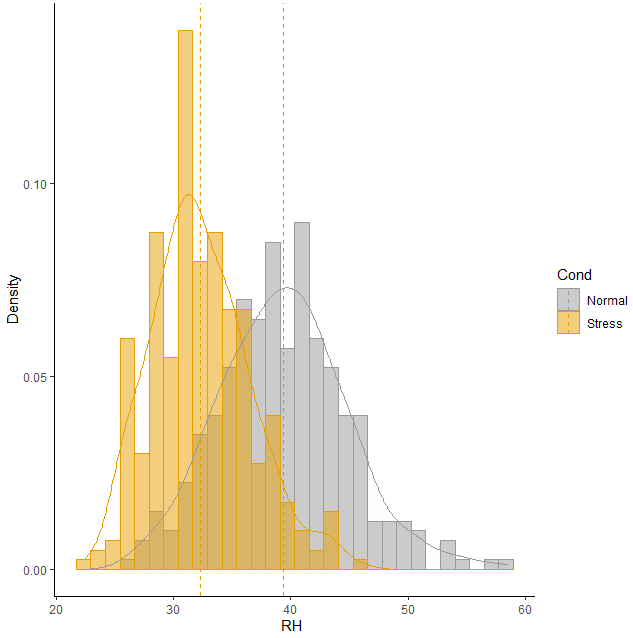 | 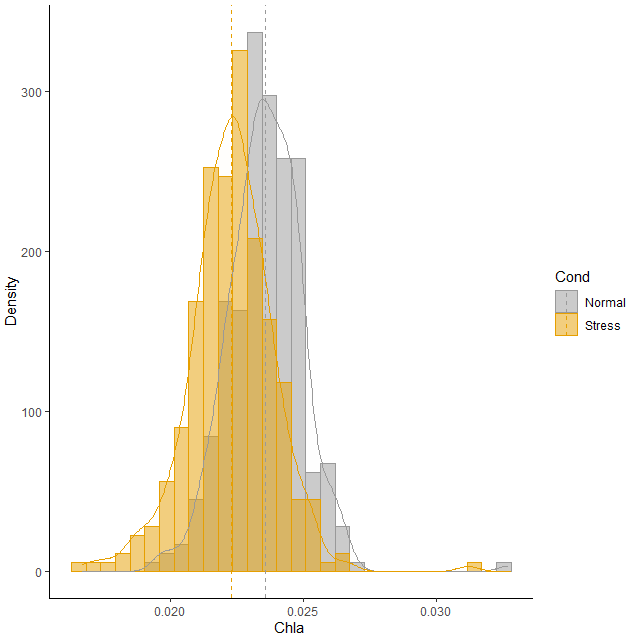 | 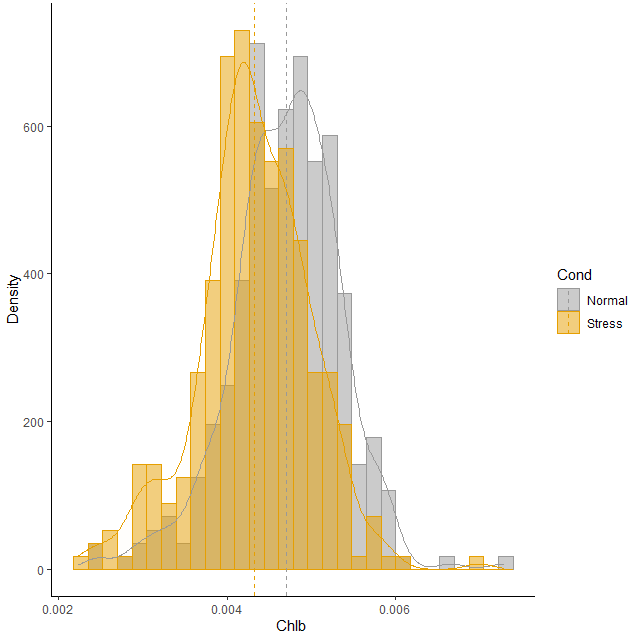 |
| --- | --- | --- |
| 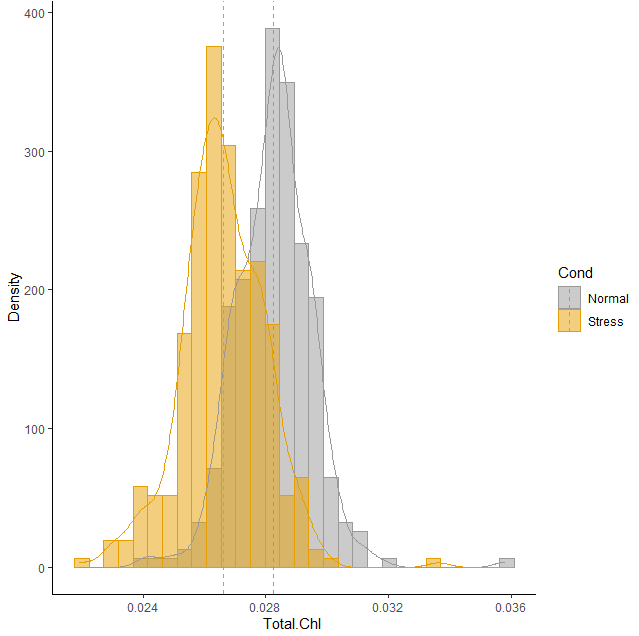 | 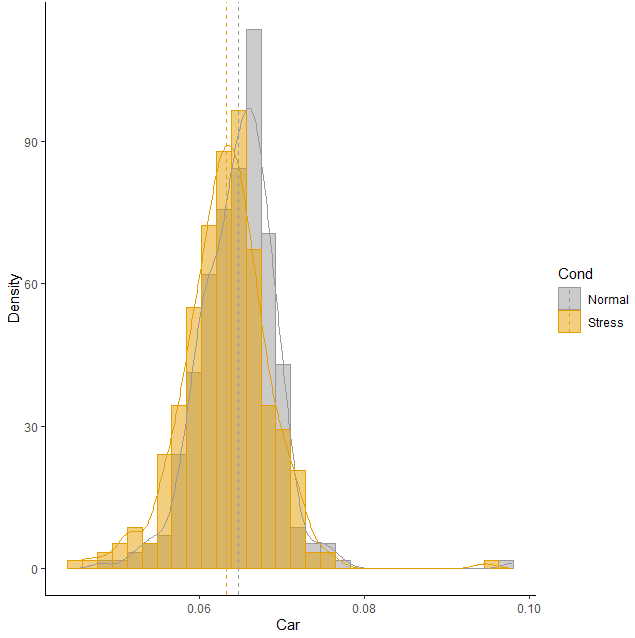 | 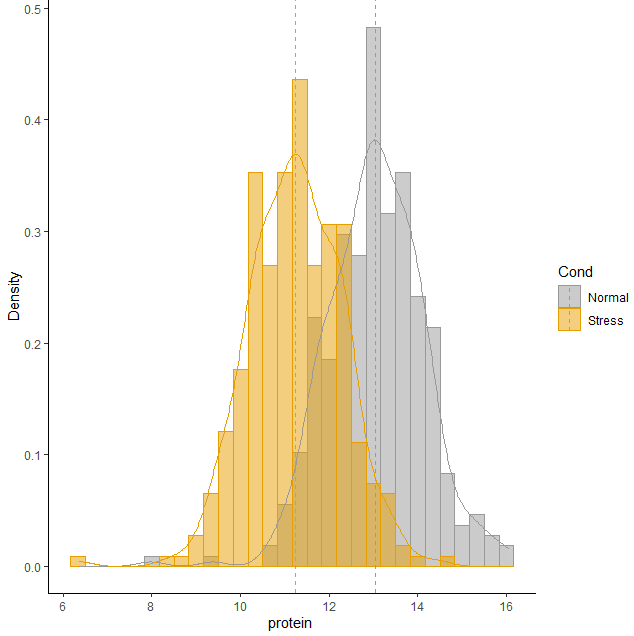 |
| 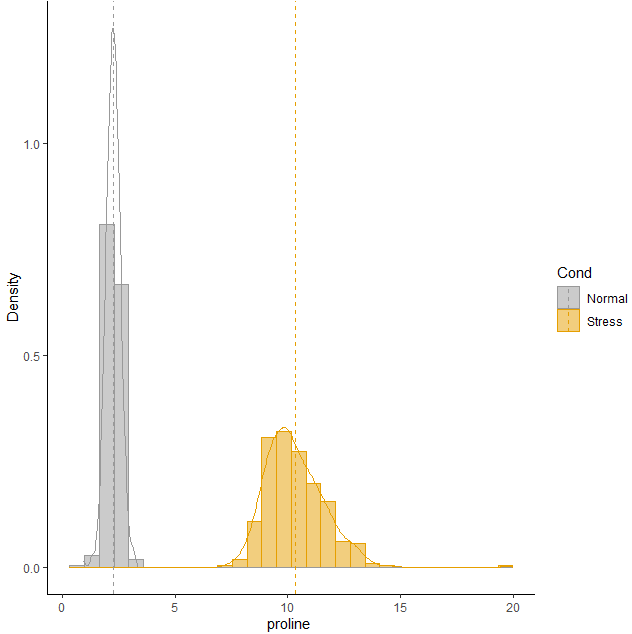 | 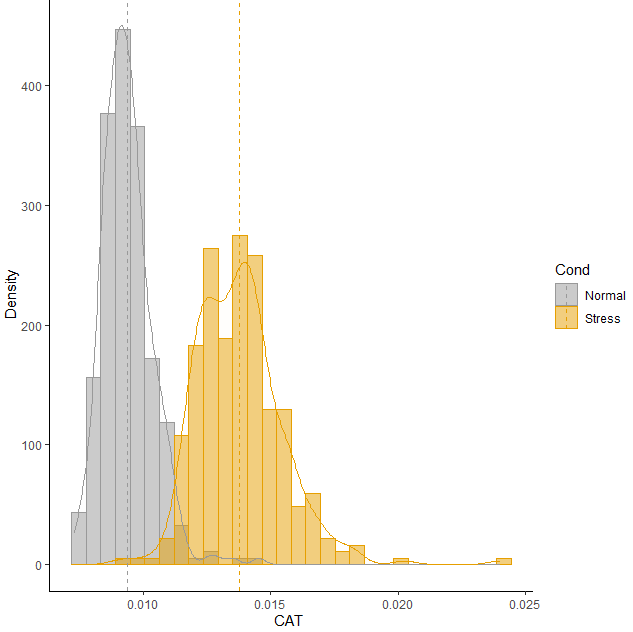 | 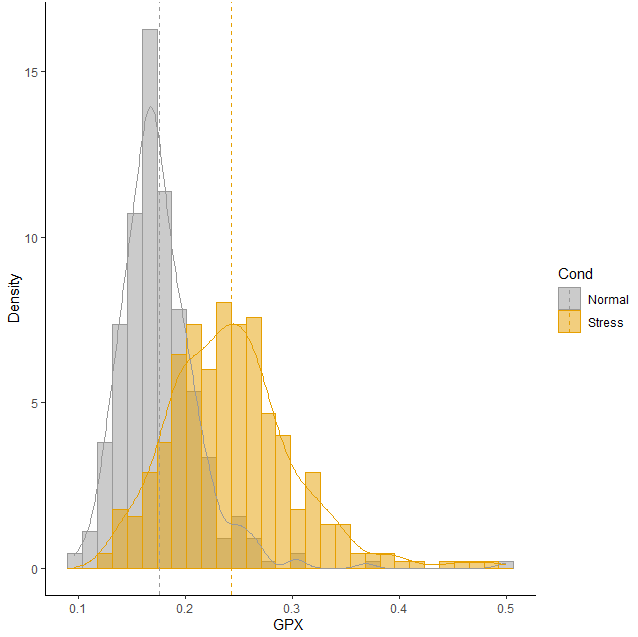 |

**Fig. 1** Continued.

| 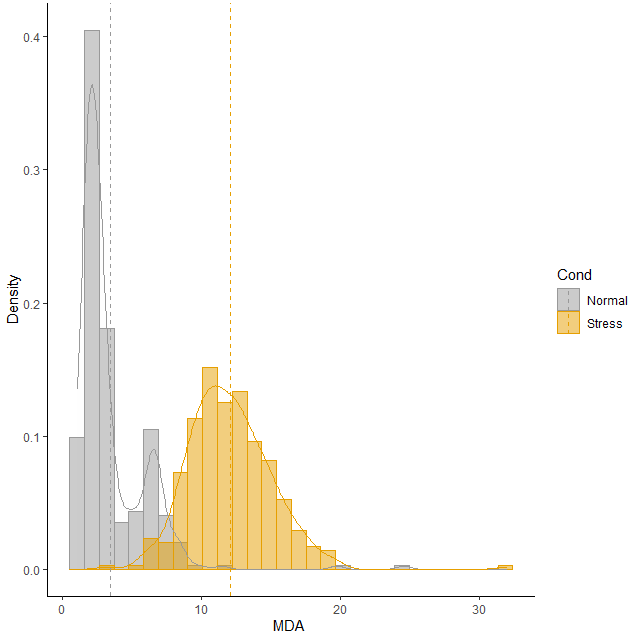 | 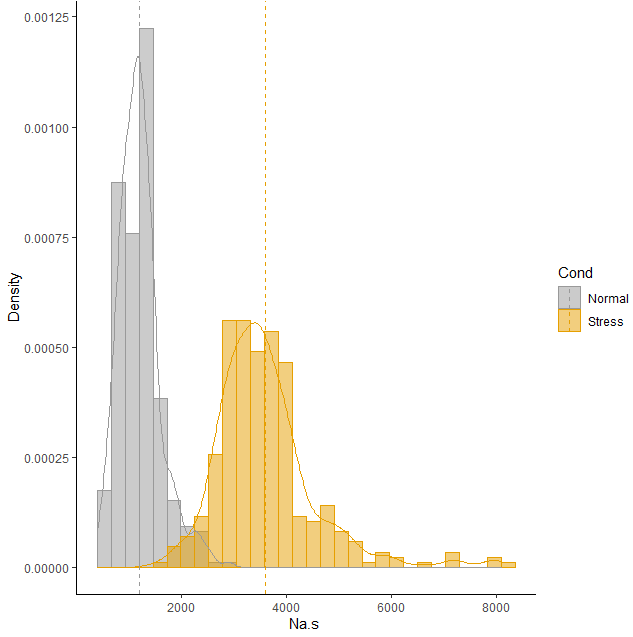 | 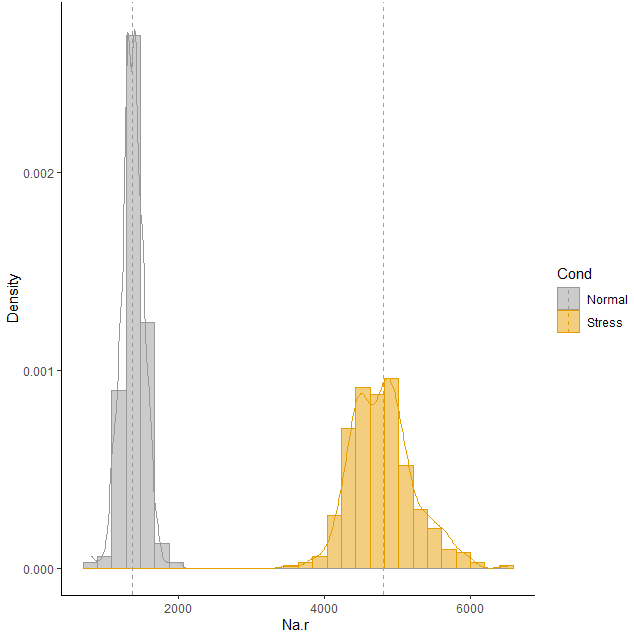 |
| --- | --- | --- |
| 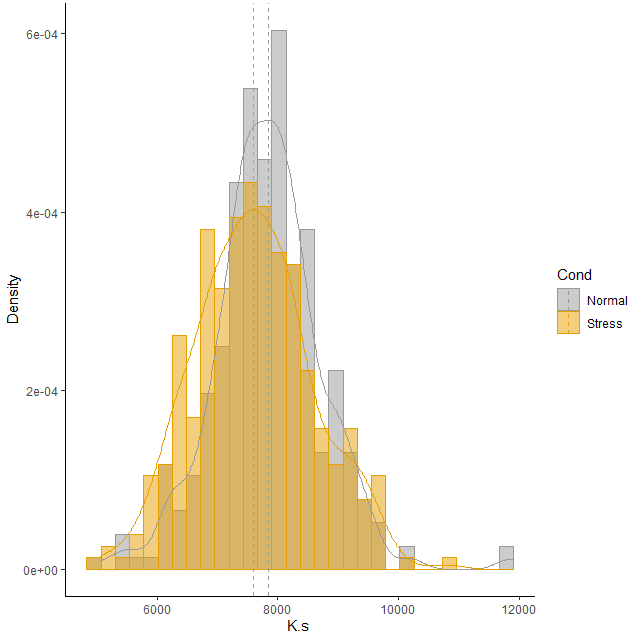 | 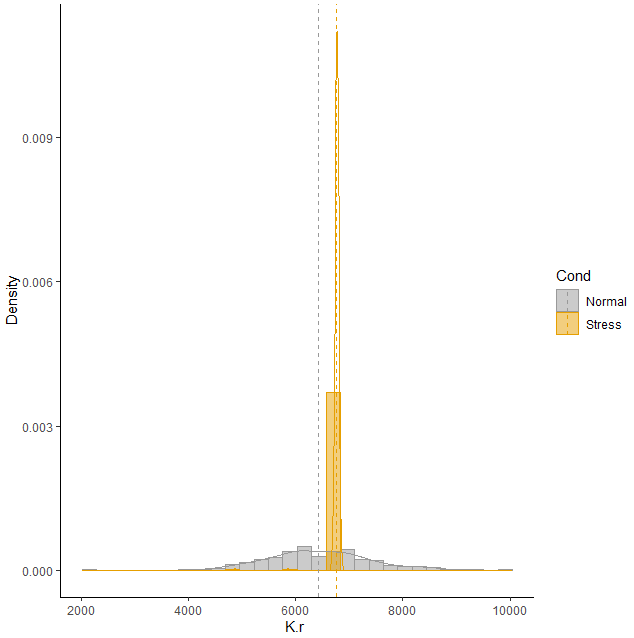 | 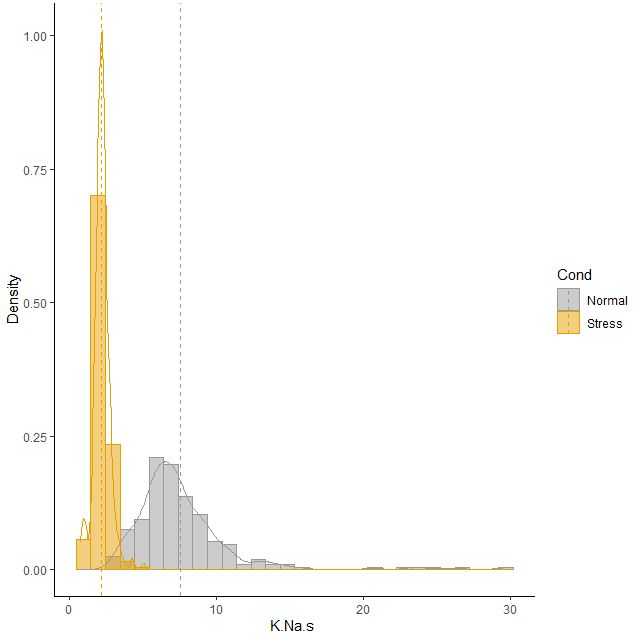 |
| 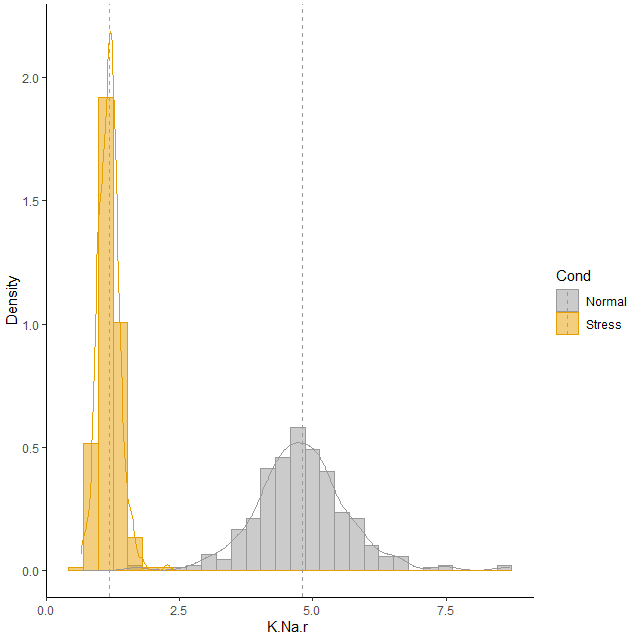 |  |  |

**Fig. 1** Continued.


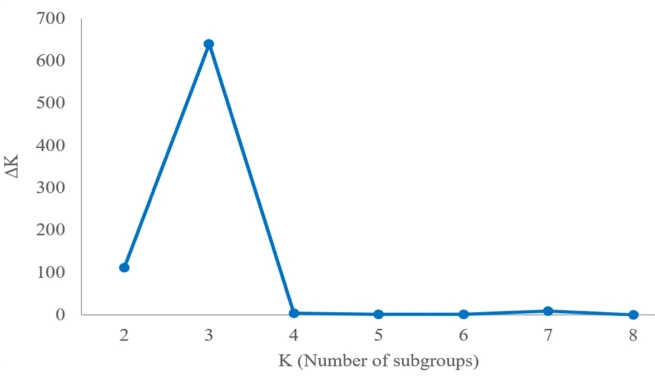


b

a


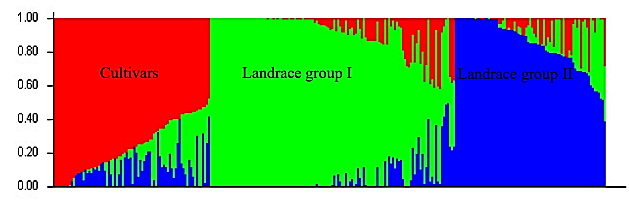


**Fig. 2S** The number of subpopulations in the wheat panel based on ΔK values (a), A structure plot of 298 wheat cultivars and landraces determined by K = 3 (b)
